# Development and external validation of the ‘Global Surgical-Site Infection’ (GloSSI) predictive model in adult patients undergoing gastrointestinal surgery

**DOI:** 10.1093/bjs/znae129

**Published:** 2024-06-21

**Authors:** NIHR Global Research Health Unit on Global Surgery and GlobalSurg Collaborative, McLean KA, Knight SR, Clark N, Ademuyiwa A, Adisa A, Aguilera-Arevalo M, Ghosh D, Haque PD, Lawani I, Medina A Ramos-De la, Ntirenganya F, Samuel S, Tabiri S, Simões JF, Shaw CA, Kamarajah SK, Picciochi M, Pius R, Pinkney T, Li E, Morton D, Nepogodiev D, Glasbey JC, Bhangu A, Harrison EM, Ademuyiwa AO, Adisa AO, Aguilera ML, Altamini A, Alexander P, Al-Saqqa SW, Borda-Luque G, Cornick J, Costas-Chavarri A, Drake TM, Fergusson SJ, Fitzgerald JE, Glasbey J, Ingabire JA, Ismaïl L, Jaffry Z, Salem HK, Khatri C, Kirby A, Kojo ATT, Lapitan MC, Lilford R, Mihaljevic AL, Mohan M, Morton D, Mutabazi AZ, Nepogodiev D, Ntirenganya F, Ots R, Pata F, Pinkney T, Poškus T, Qureshi AU, Medina A Ramos-De la, Rayne S, Recinos G, Søreide K, Shaw CA, Shu S, Spence R, Smart N, Tabiri S, Harrison EM, Bhangu A, Verjee A, Runigamugabo E, Ali THA, Rekhis S, Rommaneh M, Halhouli O, Sam ZH, Ismaïl L, Kalles V, Pata F, Nita GE, Coccolini F, Ansaloni L, Pugliesi TB, Blanco R, Gobin N, Freitas AV, Hall N, Kim S, Negida A, Khairy H, Jaffry Z, Chapman SJ, Arnaud AP, Tabiri S, Recinos G, Manipal Cutting Edge, Mohan M, Amandito R, Shawki M, Hanrahan M, Pata F, Khatri C, Zilinskas J, Roslani AC, Goh CC, Ademuyiwa AO, Irwin G, Shu S, Luque L, Shiwani H, Altamimi A, Fergusson SJ, Spence R, Rayne S, Jeyakumar J, Cengiz Y, Raptis DA, Glasbey JC, Modolo MM, Iyer D, King S, Arthur T, Nahar SN, Waterman A, Ismaïl L, Walsh M, Agarwal A, Zani A, Firdouse M, Rouse T, Liu Q, Correa JC, Salem HK, Talving P, Worku M, Arnaud A, Tabiri S, Kalles V, Aguilera ML, Recinos G, Kumar B, Kumar S, Amandito R, Quek R, Pata F, Ansaloni L, Altibi A, Venskutonis D, Zilinskas J, Poskus T, Whitaker J, Msosa V, Tew YY, Farrugia A, Borg E, Medina A Ramos-De la, Bentounsi Z, Ademuyiwa AO, Søreide K, Gala T, Al-Slaibi I, Tahboub H, Alser OH, Romani D, Shu S, Major P, Mironescu A, Bratu M, Kourdouli A, Ndajiwo A, Altwijri A, Alsaggaf MU, Gudal A, Al-Faifi JJ, Seisay S, Lieske B, Rayne S, Spence R, Ortega I, Jeyakumar J, Senanayake KJ, Abdulbagi O, Cengiz Y, Raptis D, Altinel Y, Kong C, Teasdale E, Irwin G, Stoddart M, Kabariti R, Suresh S, Gash K, Narayanan R, Maimbo M, Balmaceda R, Fermani C, Modolo MM, Chenn R, Edye M, Gobin N, Macdermid E, Yong CO, D'amours SK, Iyer D, Jarmin M, Brown J, Phillips N, Youssef D, George R, Koh C, Warren O, Hanley I, Dickfos M, Nawara C, Primavesi F, Öfner D, Hakim H, Hussain M, Kumar T, Mahmud K, Mitul AR, Oosterkamp A, Assouto PA, Lawani I, Souaibou YI, Castillo VDP, Moreira G, Munhoz MM, Careta MC, Ferreira SAK, Segundo LCB De Castro, Cury ADL, Kim SB, Sousa AV De, Fraga GP, Santos DVD, Simoes RL, Miguel GPS, Silvestre BP, Freitas AVC De, Felipe CO, Laufer LAV, Vianna JGP, Altoe F, Giuriato TF, Luiz JS, Morais PAB, Pimenta ML, Silva LAD, Araujo R, Leal A, Leal M, Menegussi J, Tatagiba LS, Lima CVB De, Chong CL, Tun AK, Aung KP, Chong CL, Yeo LS, Chong CL, Devadasar GH, Qadir MRM, Stock S, Brown J, Kabba J, Ngwa TE, Nigo S, Deckelbaum DL, Horobjowsky A, Razek T, Bailey K, Cameron B, Livingston M, Agarwal A, Azzie G, Firdouse M, King S, Kushwaha S, Zani A, D'aguzan N, Grasset E, Marinkovic B, Grasset E, Jimenez J, Macchiavello R, Guo W, Oh J, Zhang Z, Zheng F, Mendez M, Montes I, Sierra S, Arango MCM, Mendoza I, Villegas MI, Aristizã¡bal FAN, Botero JAM, Riaza VMQ, Arango MCM, Morales C, Restrepo J, Arango MCM, Cruz H, Munera A, Pezelj N, Radic M, Zamarin K, Domini E, Karlo R, Mihanovic J, Hache-Marliere M, Lemaire SB, Rivas R, Fahmy MAB, Hassan A, Khyrallh A, Shimy G, AbdelFattah I, Abdulgawad M, Abozaid M, Adel A, Al-Mallah A, Alhendy M, Baheeg M, Elgebaly A, Elshafay AE, Fattah AA, Gemeah M, Gharib A, Gharib A, Gouda A, Hanafy M, Hasan A, Kenibar A, Menshawy A, Mohammed A, Mohammed A, Osman O, Saleh O, Sayed A, Abdelkader M, Asal M, Elfil M, Ghoneem M, Gohar MEAM, Gomaa A, Gomah M, Karkeet M, Nabawi A, Rashwan H, Alahmady O, Alkammash A, Ata AAA, Attia AM, Galeel AA El, Hamid NA El, El-Dien KS, Elbanby E, Elkorashy AM, Hantour U, Kotb AHE, Mansour B, Nasr M, Saeed M, Abdel-Wahab NYE, Abozyed MAF, Adel A, Sayed GS El, Elkolaly SS, Lasheen KT, Saeed AM, Taha EMS, Youssif JH, Ahmed SM, El-Shahat NS, Khedr AEH, Afifi AM, Ebrahim OS, Metwally MM, Abbas M, Abdelraheim M, Deen KN El, Elnemr AE, Elsebaaye AO, Elzayat I, Elzayat M, Elzayyat I, Hemeda D, Khaled H, Rashad M, Salah O, Salama M, Seisa M, Tawfik G, Warda M, Elkhadrawi M, Elshaer K, Hussein A, Abdelgelil A, Abdelghany S, Aboarab A, Aboraya M, Al-Aarag AA, Kholy A El, Elbermawy M, Elkady F, Elkholy A, Elmelegy R, Elsawahly DME, Elshanwany S, Fakher R, Ghazy AA, Haroun A, Nofal E, Safa H, Sakr A, Salma M, Samih H, Samir A, Samy S, Ghanem E, Ashal G El, Shoura Y El, Hammad AM, Khairy H, Tammam A, Abdallah E, Abdelshafy M, Abouzahra A, Alzayat T, Antar S, Elfeki H, Elgendy FI, Elsheikh S, Gamaly E, Hamad MGM, Hosh M, Magdy B, Mehrez S, Abd-Elrasoul Y, Abuseif M, Alrahawy M, Ammar M, Ammar MS, Barakat SAE, El-Salam FA, Elkelany A, Elkelany A, Elrasoul YA, Elsayed N, Elwakil H, Etman M, Eysa A, Hegazy Y, Morsi M, Mustafa M, Nasr A, Raslan A, Rslan A, Saad S, Sabry A, Sadek A, Seifelnasr O, Shaker H, Toeema AG, Zidan H, Zidan H, El-Kashef H, Shaalan M, Tarek A, Almallah A, Elwan A, Elwan A, Emadeldin D, Fouad A, Ghonaim MA, Nayel AR, Sayma EA, Seif M, Hameed OSA El, El-Ma'doul AS, Elbatahgy A, Elsorogy DEAA, Lasheen A, Mosad A, Mostafa HA, Omar AA, Tolba H, Salam YA El, Ismail M, Morsi A, Abouelnasr A, Afandy A, Amer MA, Amreia M, Attallah NA, Ayad S, Magd AA El, El-Hamouly AS, El-badawy HA, Elkelany A, Elkelany A, Elsobky S, Hafez AT, Marey A, Mokhtar A, Mosalum O, Mustafa M, Sakr R, Shaker R, Shaker R, Zalabia MF, Ahmed EA, Fadel A, Mohamed MM, AlYoussef I, Aldalaq A, Ali A, Alkhabbaz D, Alnawam E, Alwafai MG, Aly AK, Dwydar A, El-Sheemy H, Kharsa S, Mamdouh E, Elashmawy M, Elazayem AA, Elkadsh I, Elsayed ZM, Elwaey A, Ghanem S, Hussein S, Meshref A, Mousa M, Nashaat A, Saad M, Darweesh M, Hafez M, Mohameden A, Badr A, Badwy A, Slam MA El, Abdelkareem A, Aboraya M, Abozeid K, Al-Nahrawi S, Allam M, Ameen M, Aql S, Dawoud H, Gendy A El, Mesery S El, Elazoul M, Eldamaty L, Elhendawy AOA, Elsehimy M, Elshobary M, Fahiem A, Hagar A, Hashish A, Hashish M, Marey AS, Nada F, Sarsik S, Shehata S, Zidan M, Badwi NM, Elfouly N, Elfouly Y, Elsherbiny AS, Fawzy A, Gheith A, Habeeb MA, Hassan A, Husseini M, Ibrahim Y, Kasem E, Mohamed O, Mohammed MMH, Rashid M, Sieda B, Soliman AR, Starr N, Worku M, Abebe NS, Desta S, Wondimu S, Asele FA, Dabessa D, Thomas E, Abebe NS, Zerihun AB, Leppäniemi A, Mentula P, Sallinen V, Alimi Q, Gaignard E, Graffeille V, Alimi Q, Gaignard E, Graffieille V, Abbo O, Bouali O, Mouttalib S, Aigrain Y, Botto N, Hervieux E, Faure A, Fievet L, Panait N, Eyssartier E, Podevin G, Schmitt F, Arnaud AP, Martin A, Parent V, Bonnard A, Muller C, Peycelon M, Frade F, Irtan S, Scalabre A, Abantanga F, Boakye-Yiadom K, Bukari M, Owusu F, Awuku-Asabre J, Bray LD, Tabiri S, Bamicha A, Lytras D, Psarianos K, Kefalidi E, Gemenetzis G, Agalianos C, Dervenis C, Gouvas N, Karousos D, Kontos M, Kouraklis G, Germanos S, Marinos C, Anthoulakis C, Mitroudis N, Nikoloudis N, Estupinian S, Forno W, Recinos G, Azmitia JRA, Cabrera CCR, Aguilera M, Guevara R, Mendez N, Mendizabal CAA, Ramazzini P, Urquizu MC, Barrios E, Barrios E, Soley R, Tale F, Mérida SMC, Rodríguez DEM, Velásquez CIP, Lopez M, Regalado F, Siguantay M, Lam FY, Leung MF, Li KKK, Li WS, Mak T, Ng S, Szeto CCL, Szeto KJ, Gyanchandani N, Kirishnan A, Prasad SS, Bhat S, Kinnera SV, Sreedharan A, Kumar BS, Rangarajan M, Kumar S, Reddy Y, Venugopal C, Mittal A, Lakshmi HN, Malik P, Nadkarni S, Jain P, Limaye N, Pai S, Khajanchi M, Satoskar R, Satoskar S, Mahamood AB, Soeselo DA, Sutanto EPR, Tedjaatmadja C, Amandito R, Mayasari M, Rahmawati FN, Al-Azraqi IAA, Al-Hameedi HII, Al-Hasani RKMJ, Ibraheem HI, Kamil R, Sabeeh L, Shawki M, Telfah MM, Gosling S, Mccarthy M, Rasendran A, Dablouk M, Dablouk MO, Gilbert RW, Hanrahan M, Kerley R, Kielty P, Marks E, Mauro L, Normile C, Rasendran A, Sheehan J, Song J, Mirghani D, Naqvi SA, Wong CS, Cahill R, Chung S, D'cruz R, Cadogan DD, Clifford C, Driscoll A, Fahy C, Gilbert R, Gosling SG, Hanrahan M, Mccarthy M, Normile C, Powell A, Rasendran A, Song J, Bowe R, Lee C, Paul S, Hanrahan M, Hutch W, Mealy K, Mohan H, O'neill M, Bondurri A, Danelli P, Maffioli A, Pasini M, Pata G, Roncali S, Carlucci M, Faccincani R, Silvani P, Khattab K, Tugnoli G, Saverio S Di, Cloro LM, Paludi MA, Pata D, Allegri A, Ansaloni L, Coccolini F, Bortolasi L, Hasheminia A, Veronese E, Benevento A, Pata F, Tessera G, Canto MD, Cucumazzo S, Nastri G, Grandinetti PP, Lamanna GL, Maniscalco A, Rausa E, Sgroi G, Turati L, Allegri A, Ansaloni L, Coccolini F, Merlini D, Monteleone M, Villa R, Cacurri A, Cirocchi R, Grassi V, Bonavina L, Ceriani C, Macchitella Y, Diab A, Elzowawi F, Waleed H, Jokubauskas M, Varkalys K, Venskutonis D, Ambrozeviciute V, Pranevicius R, Juciute S, Skardžiukaitė A, Austraite A, Bradulskis S, Dambrauskas Z, Riauka R, Urbanavicius L, Venskutonis D, Zilinskas J, Karumnas P, Urniezius Z, Zilinskiene R, Rudzenskaite A, Kaselis N, Montrimaite M, Usaityte A, Jokubonis K, Strazdas A, Jotautas V, Kolosov A, Rakita I, Beisa V, Kazanavicius D, Mikalauskas S, Poskus T, Rackauskas R, Strupas K, Beisa V, Laugzemys E, Maceviciute K, Poskus T, Strupas K, Preckailaite E, Rakauskas R, Coomber R, Johnson K, Nowers J, Das A, Periasammy D, Salleh A, Abdullah NAN, Kumar MN, Tze RGE, Kosai NR, Rajan R, Taher M, Chong HY, Goh CC, Roslani AC, Agius M, Bezzina M, Borg E, Bugeja R, Psaila J, Spina A, Vella-Baldacchino M, Colombani J, Francois-Coridon H, Tolg C, Diaz-Zorrilla C, Gonzalez SC, Medina A Ramos-De la, Jacobe M, Mapasse D, Snyder E, Osman M, Oumer R, Anyanwu L, Mohammad A, Sheshe A, Adesina A, Faturoti O, Taiwo O, Ibrahim MH, Nasir AA, Suleiman SI, Adebanjo A, Adeniyi A, Adesanya O, Atobatele K, Ogunyemi A, Oludara M, Oshodi O, Osuoji R, Williams O, Ademuyiwa A, Alakaloko F, Bode C, Elebute O, Lawal AO, Osinowo A, Adesuyi A, Adekoya A, Nwokoro C, Tade A, Ajao AE, Ayandipo OO, Lawal TA, Ali SS, Odeyemi B, Olori S, Adeniran J, Adeyeye A, Popoola A, Lossius WJ, Havemann I, Narvestad JK, Soreide K, Thorsen K, Nymo L, Wold TB, Dar M, Elsiddig M, Bhopal KF, Furqan MM, Iftikhar Z, Jawaid M, Khalique A, Nighat B, Rashid A, Zil-E-Ali A, Dharamshi HA, Faraz A, Naqvi T, Anwar AW, Anwer W, Shamsi G, Shamsi GS, Yaseen T, Yaseen TM, Aguilera O, Alvarez IIZ, Decoud HP, Delgado JM, Lohse HAS, Vega GMM, Aguilar WLM, Bautista ACM, Chiong JAC, Celis JMV, Pozo DAR, Hamasaki J, Herrera-Matta J, Temoche E, Barreda LMA, Ojeda RRB, Torres CPG, Garaycochea O, Fujii F, Mollo MC, Fã MS De, Delgado Tima Linares, Aguilar WLM, Bautista ACM, Chiong JAC, Castro MR, Jaramillo RA, Luque GB, Moran AER, Vergara ALC, Yip SBS, Basto CAA, Durand SYA, Rojas NMU, Camacho R, Huaman E, Zegarra S, Arenas A, Hinojosa C, Huaraya RC, Limache S, Lopez CE, López C, Machaca M, Pino W, Puma CMH, Rodriguez LM, Sila GM, Sila GM, Leon MZP De, Leon MZP De, Costa-Maia J, Melo R, Muralha N, Sauvat F, Dan I, Eduard P, Hogea M, Beuran M, Bratu R, Diaconescu I, Iordache F, Martian B, Vartic M, Mironescu AS, Muntean LI, Vida LC, Nsengimana VJP, Niragire A, Niyirera E, Ingabire J De La Croix Allen, Jovine E, Landolfo G, Zanini N, Alnuqaydan SA, Alomar IN, Altwigry AM, Akeel N, Aljiffry M, Alsaggaf M, Altaf A, Bakhaidar M, Habeebullah A, Khoja A, Maghrabi AA, Nawawi A, AlRowais M, Althwainy A, Osman N, Othman M, Alqahtani E, Aljohani E, Alyami R, Alzahrani M, Alhabli I, Aljohani E, Almuallem S, Alyami R, Alzahrani M, Mikwar Z, Almoflihi A, Ghandora N, Huwait A, Al-Mousa M, Al-shammari A, Adham W, Awwad S Al, Albeladi B, Alfarsi MA, Alghamdi M, Mahdi A, Altamimi A, Hassanain M, Nouh T, Aldhafeeri S, Algohary O, Sadig N, Aledrisy M, Alrifaie A, Gudal A, Alamoudi U, Alrajraji M, Shabkah A, Alghamdi B, Aljohani S, Daqeeq A, Al-Faifi JJ, Jennings V, Moore R, Ngayu N, Kong V, Connor K, Kretzmann H, Nel D, Panieri E, Sampson C, Spence R, Rayne S, Sishuba N, Carreira J, Mphatsoe AM, Tun M, Teasdale E, Wagener M, Botes S, Plessis D Du, Fernandez-Bueno F, Aguilar-Jimenez J, Garcia-Marin JA, Florez LJG, García LS, Pacheco RDA, Barneo L, Lopez-Arevalo C, Minguez G, Pagnozzi J, Quezada JHJ, Rodicio JL, Rodríguez-Uría R, Stuva JPG, Ugalde P, Herrera N, Ortega-Vazquez I, Rodriguez L, Arachchi PP, Arachchige LAJJ, Senanayake WSMKJ, Samaraweera DI, Sivaganesh S, Thanusan V, Balila RMH, Mohamed MAEH, Musa AEK, Ali H, Elabdin HZ, Hassan A, Ahmed H, Idris SAI, Mahdi S, Elsayed M, Elsayed M, Mahmoud M, Boijsen M, Lundgren P, Gustafsson U, Kiasat A, Jurdell E, Thorell A, Wogensen F, Wogensen F, Andersson L, Gunnarsson U, Sund M, Thorarinsdottir H, Utter M, Sundstrom SM, Kjellin A, Wredberg C, Frisk B, Nyberg J, Ahlqvist S, Björklund I, Cengiz Y, Royson H, Weber P, Royson H, Weber P, Borin E, Pahlsson H, Hjertberg M, Despotidis V, Schivo D, Schmid R, Deichsel F, Gerosa A, Nocito A, Eisner L, Mijuskovic B, Raptis DA, Zuber M, Breitenstein S, Schadde E, Staerkle RF, Kruspi S, Reinisch KB, Schoewe C, Novak A, Palma AF, Teufelberger G, Kimaro M, King R, Balkan AZA, Gumar M, Yavuz MA, Karabacak U, Lap G, Ozkan BB, Karakahya M, Ozkan BB, Adams R, Chang KY, Clement KD, Gratton R, Henderson L, Mcintosh R, Mcnish D, Milligan W, Morton R, Anderson-Knight H, Lawther R, Skelly B, Onimowo J, Shatkar V, Tharmalingam S, Fautz T, Woin E, Ziff O, Arman S, Arman S, Dindyal S, Gadhvi V, Talukder S, Talukder S, Chew LS, Heath J, Blencowe N, Gash K, Hallam S, Mannu GS, Snaith AC, Zachariades D, Hettiarachchi TS, Nesaratnam A, Wheeler J, Clements JM, Khan A, McCullagh D, Ahmed A, Allen JLY, Almy J, Ashton A, Deputy M, Khan T, Koumpa F, Marshall DC, Mcintyre CJ, Neophytou C, Roth J, Soon WC, Vincent J, Behar N, Jordan H, Sykes M, Rajjoub Y, Sherman T, Ardley R, Watts A, White T, Arulampalam T, Brown D, Shah A, Blower E, Gasteratos K, Sutton P, Vimalachandran D, Irwin G, Magee C, Mcguigan A, Mcaleer S, Morgan C, Braungart S, Labib P, Lafferty K, Mangan C, Mangan C, Reza L, Reza L, Tanase A, Tanase A, Gouldthorpe C, Turner M, Woodward H, Malik TAM, Proctor VK, Wild JRL, Davies J, Hewage K, Dubois A, Grant A, Mcintyre R, Sarwary S, Zardab A, Chong BFHK, Ho W, Mogan YP, Farinella E, Humm G, Tewari S, Hall NJ, Major CP, Wright NJ, Amin J, Amin J, Attard M, Baldacchino M, Burns H, Camilleri-Brennan JF, Camilleri-Brennan J, Farhad M, Jabbar A, Macdonald E, Richards J, Robertson AGN, Skehan J, Swann J, Xerri T, Xerri T, Bono P De, Bono P De, Gimzewska M, Hall TF, Mclachlan G, Giles J, Shah J, Chiu S, Chiu SMY, Highcock S, Weber B, Beasley W, Dias S, Hassan M, Maharaj G, Mcdonald R, Vlachogiorgos A, Baird A, Macdonald A, Witherspoon P, Green N, Sarmah P, Youssef H, Cross K, Rees CM, Duren B Van, Upchurch E, Abudeeb H, Hammad A, Khan K, Bowley D, Karandikar S, Karim A, Al-Obaedi O, Bhangu A, Chachulski W, Das K, Dawnay G, Ghetia M, Ghetia M, Mistry A, Richardson L, Roy S, Thompson B, Cocker DM, Prabhudesai A, Tan JJ, Ayyar S, Tyler R, Franco F Di, Gokani S, Vivekanantham S, Gillespie M, Gudlaugsdottir K, Currow C, Kim MY, Pezas T, Ali A, Atkinson K, Birring A, Das S, Edwards J, Jha M, Fozard T, Luck J, Puttick M, Ebdewi H, El-Rabaa S, Gravante G, Ibrahem AA, Salama Y, Shah R, Allott R, Bhargava A, Nnajiuba H, Chan Z, Hassan Z, Aber A, Boddy A, Dean R, Hemingway D, Makinde M, Patel V, Parakh J, Parthiban S, Hosein S, Ubhi HK, Malik K, Ward S, Alkhouri M, Barry J, Houlden C, Jennings L, Kang MK, Newton T, Bhattacharya S, Farquharson A, Raza I, Chang K, Henderson L, Milligan W, Blundell R, Chan E, Ibrahim I, Lim PJ, Neo YN, North AS, Peck FS, Williamson A, Wilson MSJ, Fouad D, Minocha A, Chambers A, Court E, Mccarthy K, Beaton C, Tham JC, Yee J, Bokhari S, Griffiths M, Howells L, Lockey J, Walsh U, Yallop L, Jackson P, Nasher O, Singh S, Fozard T, Luck J, Puttick M, Ho WC, Pabla G, Shariffuddin AM, Wilson MS, Doughty J, Ramzi S, Zeidan S, Davenport R, Lewis J, Sinha S, Duffy L, Mcaleer E, Williams E, Boal M, Brogden T, Griffiths E, Harrison N, Javed O, Nepogodiev D, Tafazal H, Clark DJ, Glover TE, Obute RD, Javed O, Som R, Akhtar M, Boshnaq M, Capleton P, Doughan S, Mohamed I, Rabie M, Brown E, Dempster E, Dickson L, Garland A, Kennedy M, Maple N, Monaghan E, Samuel D, Wolf B, Anderson D, Anderson R, Mcphee A, Hassan S, Smith D, Sutton P, Boereboom C, Lund J, Murphy J, Tierney G, Tou S, Daniels I, Findlay-Cooper K, Stasinou T, Smart NJ, Warwick AM, Zimmermann EF, D'Souza R, Mitrasinovic S, Omara S, Ray S, Varcada M, Hanks A, Parkinson L, Spurr M, Abington E, Ma J, Ramcharn M, Williams G, Kennedy ED, Winstanley J, Yeung ENW, Fairfield C, Fairfield C, Fergusson SJ, Jones C, Koh S, Liew I, Lim SJ, Nair H, O'neill S, Oh J, Wilson A, Anandkumar D, Ashraf SF, Basson S, Chandrakumar C, Fowler AJ, Jones TF, Kirupagaran A, Lakhani SM, Mclean AL, Patel P, Torrance HD, Batt J, Benons N, Bowman C, Stoddart M, Harrison R, Mason C, Quayle J, Barker T, Harper E, Summerour V, Hampton M, Smith C, Drake TM, Heywood EG, O'Connor T, Pitt SK, Ward AE, Chowdhury A, Hossaini S, Watson NF, Chun A, Farah A, Mckechnie D, Koh H, Lim G, Sunderland G, Browning DRL, Munipalle PC, Rooney H, Chambers A, Gould L, Decker E, Giuliani S, Nemeth K, Pereira B, Shalaby A, Chen CY, Chhabra S, Chidambaram S, Kulasabanathan K, Szczap A, Benger M, Choi J, Khalili M, Patel K, Sheth S, Singh P, Palkhi EYA, Shaikh S, Tan CY, Barnacle J, Harbord P, Kostov E, Macfarlane A, Marples L, Thurairaja R, Baillie K, Hafiz S, Palliyil MM, Porter J, Raslan C, Saeed M, Soltani N, Zikry M, Boyce T, Jones E, Whewell H, Robertson N, Th'ng F, Galloway S, Mirza A, Saeed H, Afzal M, Elena G, Zakir M, Clark T, Hand C, Holton P, Livesey A, Sodde P, Sriram A, Bharj IS, Iqbal FM, Sinha Y, Jenvey C, Rotundo A, Slade R, Abdullah AAN, Donoghue D, Giacci L, Golding D, Haines S, Harrison P, Loughran D, Sherif MA, Tang A, Tilston TW, Kotecha D, Acharya P, Chapman A, Elshaer M, Riaz A, Shalhoub J, Urbonas T, Grossart C, McMorran D, Hawkins W, Loizides S, Mlotshwa M, Ho CW, Krishna K, Orchard M, Howie EE, Khan S, Shukla J, Taylor F, Thomson P, Komolafe O, Macdonald L, Mcintyre N, Cragg J, Parker J, Stewart D, Farooq T, Lintin L, Tracy J, Kaafarani H, Luque L, Molina G, Beyene R, Sava J, Scott M, Kennedy R, Swaroop M, Azodo IA, Chun T, Heffernan D, Stephen A, Punja V, Sion M, Weinstein MS, Bugaev N, Goodstein M, Razmdjou S, Hemmila M, Napolitano L, To K, Etchill E, Kesinger M, Puyana JC, Hoogakker E, Jenner E, Todd O, Galiqi G, Grizhja B, Ymeri S, Balmaceda R, Carmona JM, Fermani CG, Modolo MM, Villalobos S, Antezana D, Aviles S, Beleño AEM, Costa C, Klappenbach R, Sanchez B, Cox D, Deutschmann P, Hamill D, Sandler S, Ashtari M, Franco H, D'Amours S, Iyer D, Niranjan N, Ljuhar D, Nataraja R, Sharpin C, Gray D, Haines M, Amin S Al, Alamin S, Karim R, Roy S, Tori SA, Faruq A, Haque M, Iftekhar F, Kanta TH, Razzaque J, Salma U, Karim S, Mitul AR, Aman NF, Estee MM, Jonnalagadda R, O'Shea M, Padmore G, Khokha D, Khokha V, Filatau A, Litvin A, Paulouski D, Shachykava T, Shubianok M, Djivoh F, Dossou F, Gbessi DG, Ismaïl L, Noukpozounkou B, Seto DM, Souaibou YI, Hodonou F, Keke KR, Ahounou EYS, Alihonou T, Ahlonsou G, Dénakpo M, Bedada AG, Barendegere V, Kwizera S, Nsengiyumva C, Choi P, Stock S, Agarwal A, Azzie G, Firdouse M, Jamal L, Kushwaha S, Zani A, Chen T, Yip C, Montes I, Sierra S, Zapata F, Arango MCM, Lanau MIV, Restrepo IM, Arango MCM, Giraldo RSR, Sierra S, Domini E, Karlo R, Mihanovic J, Abdelaziz MA, Gado A, Hantour U, Ibrahim AM, Ibrahim K, Abd-Elmawla M, Abdelkader M, Aboul-Naga MS, Adam N, Ahmed LAM, Alkelani M, Allam M, Alnaby MH, Assal A, Ebidy M, Ebidy MM, Gendy NH El, El-Din RA, Elbisomy KH, Elgendy AH, Elrazek AA, Elsawy A, Elsharkawy AA, Fahim M, Hamed MF, Hassanein AB, Ismail A, Ismail M, Karkeet M, Mabrouk M, Magdy E, Mahmoud MI, Mamdouh R, Moghazy ME, Mohamed M, Mowafy B, Nazir M, Shakshouk HAG, Shalaby M, Sleem M, Zahran D, Abdelhady S, Aboelsoud MR, Adel I, Ahmed H, Anwar N, Arafa O, Asar YH, Awad SA, Elsabbagh N, Elsherif FA, Gadelkarim M, Gamal S, Ghoneim O, Hany E, Hesham O, Hilal K, Hossameldin A, Ibrahim M, Morshedy EM, Omar ME, Rida AHEF, Saad R, Salama M, Salem M, Soliman N, Aamer A, Abdelraouf AM, Abdelshakour M, Azizeldine MG, Bassit KA, Dahy A, Hasan A, Hashim A, Ibrahim A, Mahmoud B, Mahmoud MA, Mohamed B, Qenawy M, Rashed AM, Saad MM, Sabour FA, Sayed F, Sayed M, Shamsedine AW, Shawqi M, Attia A, El-Dien KS, Shwky A, Abdel-Kader SM, Abdelaty M, Abdulaziz H, Abdulhakeem EM, Abdullah N, Abouzaid A, Abubakr M, Alaael-Dein S, Ali E, Amin HAA, Sayed IM El, El-Din SA, Eldeen EA, Eldin MAB, Elhusseiny AAE, Elsayed NAR, Elshaar M, Gamil D, Hashad E, Ibraheem AAF, Ismail MK, Madkor MH, Magdy H, Mahmoud SME, Mansour S, Mohamed AR, Mohamed F, Mohamed MA, Ramadan MT, Reda A, Refaat A, Saami M, Salah OM, Salem MM, Shawky MY, Soliman NA, Sroor F, Talaat M, Tarek A, Zakaria M, Loaloa MR, Ahmed S, Ali A, Badawy M, El-Sagheer N, Essam A, Gamal D, Magdy S, Salah A, Salah M, Abdelaal A, Aglan A, Ali S, Ata A, Darwish AKZ, Halawany M El, El-Gizawy E, Elazab A, Elhadry S, Elhalawany E, Elmihy S, Essam M, Farag A, Hajeh H, Moussa O, Nashat M, Nasr M, Rezq A, Sallam AE, Samy M, Samy M, Sheta A, Soliman S, Tariq S, Zohair A, Abdel-Aty A, Abdelhamed R, Abdelkader O, Ashour K, El-Taher E, Elhadad A, Farouk SAM, Ghanem S, Hassaan A, Ibrahim EM, Matter SM, Mohamed A, Rakha I, Soliman Y, Tarek D, Abdelazeam AR, Adelshone A, Adnan AB, Al-Marakby D, Ali CDM, Amreia M, Ata AY, Bahar S, Basir ERM, Elhendawy A, Hasnan MB, Ismail MJB, Kamarulzamil SNA, Latif A, Lokman MAA, Majid AHHA, Salma M, Shaharuddin S, Zulkifli A, Abdelbadeai K, Abdelfatah A, Abdullah MA, Ahmed H, Allam Y, Arafa S, Badwi NM, Dahab AA El, El-Sehily A, Elfouly N, Elfouly Y, Elhoseny G, Elkhalek EA, Ezzat E, Ezzat T, Fathy AM, Fergany A, Hassaan A, Hassan ATA, Hassan OMM, Ibrahim A, Ibrahim A, Kasem EA, Kelany M, Magdy M, Mohamed A, Mohammed AR, Mohammed MM, Mohammed S, Reda A, Saad AG, Saad HA, Sleem AS, Zakaria Y, Abdelazim G, Abdelmotaleb I, Abdrabou AK, Aboelella M, Aboelmagd O, Adel BE, Ahmed A, Ahmed S, Meligy A Al, Alhady K, Aly MYM, Bakry HM, Bassem M, Bekhet AH, Bekhet NM, Dabbour K, Dawood K, Kashash A El, El-Latif NKA, Elhadary NM, Elhelbawy MS, Elkholy SS, Elnagar A, Elnajjar MA, Elsameea AA, Elsherbiney S, Elzahed N, Emadeldin H, Essam AA, Gaafar S, Gad A, Gad MO, Geuoshy A, Hafez M, Hafez S, Hamsho W, Hasan D, Hassan I, Husseiny R, Ismail SA, Kandil AM, Magdy A, Maher ME, Mahmoud H, Mahmoud S, Maraie N, Mattar O, Mesbah N, Mohamed SR, Saad H, Sabe A, Sabe AK, Saeed M, Saleh AA, Semeda N, Shahine A, Soliman A, Tawfik BA, Wael N, Zakaria E, Abdallah E, Abdel-Hameed N, Denewar A, Elashry R, Elfeki H, Emara E, Emile S, Ghanem A, Mostafa M, Omar MFW, Rashad E, Sakr A, Sanad A, Tawfik G, Thabet W, Youssef M, Zaki A, Abdelmageed E, Abdelrouf DM, Raouf EA Al, Elbanby ES, Elfarargy A, Elgheriany M, Elhamouly S, Elmasry M, Elwy E, Esam A, Farahat MM, Gamal E, Gamal H, Hammad A, Hegazy EM, Ibrahim E, Kandil H, Khafagy T, Khallaf S, Mansor EY, Moaty M, Mohamed AM, Mohammed AE, Moustafa A, Nagy GS, Saidbadr A, Eid MM, Eldafrawy M, Eldeeb AZ, Rafati AAR Al, Badr MFM, Bakr A, El-Sawy A, Elsemelawy R, Mostafa M, Attar SM Al, Badenjki MA, Soliman A, Reinsoo A, Saar S, Talving P, Fitsum A, Seyoum N, Worku T, Leppäniemi A, Sallinen V, Tolonen M, Delforge X, Haraux E, Mariani A, Podevin G, Schmitt F, Haffreingue A, Marret J, Rod J, Bustangi N, Lopez M, Scalabre A, Bréaud J, Gastaldi P, Lecompte J, Ballouhey Q, Fourcade L, Grosos C, Cecilia T, Helene F, Jean-Francois C, Grella MG, Arnaud AP, Courboin E, Hascoet J, Maillot B, Renaux-Petel M, Abbo O, Kaci AA, Prudhomme T, Dousset B, Gaujoux S, Schiavone R, Dardenne S, Robert E, Broch A, Hervieux E, Muller C, Anis E, Claire R, Taieb C, Irtan S, Parmentier B, Peycelon M, Akatibo E, Ekow M, Yakubu M, Gyamfi FE, Atkins ET, Coompson CL, Amoako-Boateng M, Dayie M, Debrah S, Hagan R, Ackom E, Akoto E, Mensah E, Kwakyeafriyie P, Asare-Bediako K, Kordorwu HEK, Tackie E, Adu-Aryee N, Amoako J, Appeadu-Mensah W, Bediako-Bowan A, Bonney W, Clegg-Lampety J, Dakubo J, Dedey F, Essoun S, Etwire V, Glover-Addy H, Ohene-Yeboah M, Osei-Nketiah S, Agbedinu K, Amoah M, Dally C, Gyedu A, Yifieyeh A, Amoako PT, Dagoe E, Owusu F, Abantanga F, Appiah EK, Asumah H, Bandoh D, Kojo ATT, Kyereh M, Tabiri S, Wondoh P, Aaniana K, Acquah E, Avoka A, Kusi K, Maison K, Opoku-Agyeman R, Dassah V, Davor A, Abdul-Latif S, Barnabas GN, Gkiokas G, Papailia A, Theodosopoulos T, Ioannidis O, Kyziridis D, Parpoudi S, Bamicha A, Lytras D, Psarianos K, Gemenetzis G, Parasyris S, Farmakis K, Feidantsis T, Mitroudi M, Panteli C, Patoulias I, Sfougaris D, Valioulis I, Karabelias G, Kyrou G, Papaskarlatos I, Germanos S, Konstantina K, Zampitis N, Stefanopoulos A, Agalianos C, Barkolias C, Ferousis C, Ivros N, Kalles V, Kyriazanos I, Tselos A, Tzikos G, Voulgaris E, Balalis D, Korkolis D, Manatakis DK, Anthoulakis C, Margaritis M, Nikoloudis N, Aguilera-Arevalo M, Coyoy-Gaitan O, Rosales J, Cohen DM, Matheu A, Rosenberg GS, Cruz DH, Galvez CP, Rodriguez STT, Barrios E, Soley R, Tale L, Charles A, Paul M, Chan TK, Cheung YHE, Dao W, Fok CYJ, Kwok SH, Lai AC, Lam JCY, Lam WH, Lee TSB, Leung KW, Li KHG, Mak TWC, Ng YK, Wong HY, Yeung MHA, Foo CC, Liu Q, Yang J, Kumar S, Alexander P, Aruldas N, Dar W, Janardha KC, Muddebihal U, Bhatnagar A, Kumar B, Upadhyaya V, Adella FJ, Iskandar F, Rulie AS, Setiawan J, Evajelista CV, Natalie H, Suyadi A, Adhitama N, Andika FFA, Arsyad HM, Gunawan R, Hasanah A, Karismaningtyas H, Mata LPS, Mukin ADF, Nurqistan HD, Purwaningsih NA, Rahmah DF, Widiastini TA, Amandito R, Billy M, Clarissa A, Gultom PA, Haloho A, Jeo WS, Johanna N, Lee F, Sutandi N, Alherz M, Conlon KC, Dorani RMNR, Glynn M, Goh W, Shiwani HA, Sproule L, Bala M, Kedar A, Armellini A, Chiesa D, Pata G, Ansaloni L, Coccolini F, Nita GE, Vicario E, Confalonieri G, Pesenti G, Brunoni B, Rinaldi A, Ringressi MN, Bortolasi L, Campagnaro T, Conci S, Gulielmi A, Iacono C, Lazzari G, Manfreda S, Tedeschi U, Violi P, Ciccioli E, Goldin E, Vendramin E, Aquilino F, Chetta N, Picciariello A, Andreotti D, Gavagna L, Occhionorelli S, Targa S, Vasquez G, Basso SMM, Bigaran A, Favero A, Migliore M, Mochet S, Perino M, Riente F, Salusso P, Sasia D, Clerico G, Gallo G, Trompetto M, Papandrea M, Sacco R, Sammarco G, Bucci L, Giglio MC, Luglio G, Pagano G, Peltrini R, Sollazzo V, Foco M, Giardino FR, Gui D, Perrotta G, Ripa M, Pasquali S, Simioni A, Boni D De, Bonavina L, Lazzari V, Macchitella Y, Abdelkhalek M, Belli A, Franciscis S De, Birindelli A, Tugnoli G, Saverio S Di, Mingrone P, Paludi MA, Pata D, Basilicò S, Corbellini C, Merlini D, Bondurri A, Leone N, Maffioli A, Aonzo P, Curletti G, Galleano R, Brocca AL, Cocorullo G, Falco N, Fontana T, Licari L, Mangiapane M, Salamone G, Silvestri V, Tutino R, Marco P De, Arcudi C, Shalaby M, Sileri P, Angelieri D, Antoniozzi A, Basso CD, Catani M, Coletta D, Coletti M, Depalma N, Falaschi F, Iannone I, Malavenda M, Natili A, Reali C, Ribaldi S, Rossi D, Berti S, Boni S, Francone E, Benevento A, Giavarini L, Pata F, Balducci G, Conte AL, Lorenzon L, Bianco F, Steccanella F, Turati L, Pellino G, Selvaggi F, Selvaggi L, Martino N Di, Ababneh A, Abusalem L, Al-Dakka E, Aljboor K, Alnusairat A, Bsisu I, Halhouli O, Qaissieh A, Mohammed H, Yusufali T, Lando J, Ndegwa W, Parker R, Dragatas D, Višinskas P, Žilinskienė R, Dulskas A, Kuliavas J, Samalavicius NE, Gribauskaite J, Jokubauskas M, Venskutonis D, Bradulskis S, Dainius E, Dambrauskas Z, Gulbinas A, Jankus T, Jasaitis K, Kasputyte S, Kiudelis M, Mikuckyte D, Montrimaite M, Nevieraite V, Parseliunas A, Petrikenas S, Riauka R, Slapelyte E, Subocius A, Venclauskas L, Zilinskas J, Kaselis N, Žiubrytė G, Pažuskis M, Urniežius Z, Vilčinskas M, Burmistrovas A, Tverskis Z, Mazelyte R, Vaicius A, Zadoroznas A, Abaliksta T, Banaitis VJ, Danys D, Drungilas M, Gaižauskas V, Grisin E, Jotautas V, Ladukas A, Lagunavicius K, Laugzemys E, Lipnickas V, Majauskyté D, Mazrimas P, Mikalauskas S, Poškus T, Rackauskas R, Simutis G, Sruogiene EZ, Uščinas L, Rahantasoa FCFP, Rasoaherinomenjanahary F, Samison LH, Tolotra TEC, Kwatiwani C, Msiska N, Msosa V, Mukuzunga C, Asilah SMD, Chai FY, Gunaseelan K, Nasir WN'WM, Syibrah KZ, Yoganathan P, Koh PY, Lee EX, Lim SY, Saw JE, Teo SY, Yeang LJ, Gan YY, Ting JRS, Cheah AEZ, Chow CYN, Der Y, Har PAL, Koay KL, Mat TNT, Sii SSY, Tan YK, Wong CY, Cheong YJ, Gan C, Heng HE, Kong SN, Mok YT, Neo YT, Palayan K, Tan YW, Tata MD, Chin PX, Riswan NZ, Salleh A, Abdullah NAN, Ali SAWEW, Chung KJ, Jethwani DL, Julaihi R, Mathew SW, Nirumal MK, Tze RGE, Yahaya MT, Henry F, Low X, Tew YY, Aziz DNA, Kosai NR, Rajan R, Taher MM, Aziz NA, Chai C, Chong H, Kumar S, Poh K, Roslani AC, Bertuello I, Bonavia K, Borg E, Brincat SD, Camilleri GM, Carabott K, Cassar K, Dalli J, Dimech T, Falzon M, Farrugia A, Grech N, Grima T, Le VTH, Magri D, Mizzi C, Mizzi S, Navarro A, Sammut K, Scicluna R, Shaikh N, Tembo T, Zammit S, Zarb C, Corro-Diaz S, Manriquez-Reyes M, Medina A Ramos-De la, Abbouch M, Abdelhamid A, Bachri H, Belkouchi A, Benammi S, Bennai RM, Benyaiche C, Boukhal K, Hrora A, Jabal MS, Duinhouwer L, Vermaas M, Merlo MS, Pastora J, Wood G, Adamu A, Aliyu H, Aliyu M, Aliyu S, Baba S, Daniyan M, Ogunsua O, Sholadoye T, Ukwenya Y, Anyanwu L, Mohammad A, Sheshe A, Adebola O, Adesina A, Faturoti O, Odutola O, Onuoha C, Taiwo O, Ajah J, Kache S, Makama J, Abiola O, Adeyeye A, Ajiboye A, Amole I, Olaolorun A, Adebanjo A, Adeniyi A, Adesanya O, Ajai O, Balogun F, Njokanma I, Oludara M, Osuoji R, Williams O, Ademuyiwa A, Adenekan B, Alakaloko F, Bode C, Elebute O, Ihediwa G, Lawal A, Nwinee V, Olajide TO, Olugbemi M, Oshati O, Osinowo A, Abdurrazzaaq A, Ajao A, Ayandipo O, Lawal T, Mshelbwala P, Odeyemi B, Olori S, Samson G, Samuel SA, Timothy OK, Adeniran J, Adeyeye A, Alada M, Habeeb O, Nasir A, Popoola A, Bello B, Mendel H, Muktar U, Augestad KM, Banipal GS, Moe TT, Monteleone M, Schultz JK, Gaarder T, Monrad-Hansen PW, Næss PA, Herikstad R, Kanani A, Larsen JW, Styles K, Søreide JA, Søreide K, Veen T, Holte S, Lauzikas G, Wiborg J, Aahlin EK, Gran M, Jensen E, Abbasy J, Alvi AR, Gala T, Shahzad N, Nadeem N, Saqlain M, Ahmed A, Bhopal KF, Butt MT, Iftikhar Z, Niazi AK, Razi SAU, Javaid M, Khan MA, Waqar M, Adil M, Baluch F, Bani-Sadar A, Qureshi AU, Raza A, Raza A, Raza I, Amjad M, Arshad MM, Abushamleh S, Al-taher T, Hamarshi A, Hamdan A, Hanoun S, Jaradat D, Musleh A, Qumbos AA, Saadeh R, Salman A, Taher AA, Al-farram H, Al-saqqa S, Awad I, Bowabsak A, Jamassi A El, Firwana A, Hamdan M, Hasanain D, Salah M, Altarayra M, Ghannam M, Herebat A, Qawasmi I, Qurie K, Shaheen A, Adawi I, Adawi M, Elmashala A, Barrawi FE Al, Ashour A, Ghaben A, Ashour A, Abuowda Y, Afana S, Al-Buhaisi A, Alaloul E, Alyacoubi S, Baraka H, Elshami M, Jaber S, Meqbil J, Khreishi R, Khreishi R, Abuqwaider E, Idress T, Al-faqawi M, Al-khatib A, Fares M, Abdelhaq A, Abu-toyour M, Asi F, Atiyeh A, Dabboor M, Mustafa M, Shalabi A, Shamasneh A, Zaa'treh R, Cardozo JT, Cardozo RAM, Lohse HAS, Lopez LIP, Roche MO, Servin GRP, Vega GMM, Salcedo J, Velasquez R, Barrantes AMS, Bravo JAC, Dueñas CG, Espinoza KT, Fernández C, Fuentes-Rivera L, Málaga B, Romani D, Shu S, Ye J, Barrientos LAM, Farfan ESF, Hamaguchi JLH, Matta JJH, Robledo-Rabanal A, Solis LAZ, Velásquez AJR, Bermúdez YEA, Calua AC, Carpio J, Carrasco N, Espinoza F, Miyasato HS, Orbegozo PAT, Ortiz N, Panez WR, Razuri C, Rodriguez X, Rojas ADP, Samaniego CS, Sanchez D, Saravia F, Torres SG, Valcarcel-Saldaña M, Contreras-Vergara AL, Mejia AGV, Montejo MSG, Espinoza KT, Mas R, Paucar ADP, Salas MDCE, Sila GCM, Ticona WA, Vargas M, Almanon CL, Lapitan MC, Parreno-Sacdalan MD, Maño MJB, Mora JJV, Redota MAP, Roxas MF, Lasek A, Major P, Radkowiak D, Rubinkiewicz M, Janik M, Roszkowski R, Walędziak M, Costa-Maia J, Fernandes C, Melo R, Beuran M, Bratu MR, Ciubotaru C, Diaconescu B, Negoi I, Vartic M, Kourdouli A, Popa M, Mironescu AS, Muntean L, Vida LC, Mircea H, Duhoranenayo D, Ingabire JCA, Mutabazi AZ, Uzabumwana N, Jovine E, Landolfo G, Zanini N, Alghamdi MSA, Aljiffry M, Alkaaki A, Altaf A, Idris F, Khoja A, Maghrabi A, Nawawi A, Turkustani S, Jeremic L, Nestorovic M, Radojkovic M, Chan XW, Chong CS, Joel LWL, Koh S, Law JH, Lee KY, Lee KC, Leong FQH, Lieske B, Tan JK, Tan KSK, Tan RCK, Maistry N, Jennings V, Leusink A, Moore R, Mabitsela ME, Ndlovu SR, Kong V, Joosten J, Pape J, Roodt L, Sander A, Sobnach S, Spence R, Rayne S, Straten S Van, Anderson F, Madiba T, Moodley Y, Kinandu K, Ndwambi P, Tun M, Plooy F Du, Badicel M, Jaich R, Chilton G, Hartford L, Karjiker P, Bougard H, Chu K, Dell A, Gouws J, Kariem N, Noor F, Kabongo K, Khamajeet A, Tshisola SK, Burger S, Ellison Q, Grobler DC, Khulu LB, Toit F Du, Dedekind B, Hampton MI, Nashidengo P, Pluke K, Bernardo CG, Contreras E, Dorismé A, García LS, Pagnozzi J, Rodicio J, Sanz S, Stuva J, Suarez A, Vico TD, León AM De, Garcia-Florez L, Otero-Díez JL, Pérez VR, Suárez NA, Ambrona-Zafra D, Craus-Miguel A, Diaz-Jover P, Fernandez-Vega L, Garcia-Perez JM, Jimenez-Morillas P, Mazzella A, Pineño-Flores C, Pujol-Cano N, Segura-Sampedro JJ, Sena-Ruiz F, Soldevila-Verdeguer C, Carneros VJ, Collado MV, García JM, Moreno SC, Septiem JG, Andriola V, Blanco-Colino R, Espin-Basany E, Esteban E, Ferrero E, Gonzalez M, Ortega I, Picardo A, Ruiz-Tovar J, Jayathilake AB, Thalgaspitiya SPB, Wijayarathna LS, Wimalge PMS, Ndajiwo A, Okenabirhie O, Sanni HA, Abdulaziz M, Adam A, Homeida A, Mussad A, Omer OA, Younis A, Hjertberg M, Thorell A, Wogensen F, Thorarinsdottir H, Elbe P, Forlin L, Rutkowski W, Saraste D, Breistrand M, Sokratous A, Ahlqvist S, Ahlqvist S, Björklund I, Cengiz Y, Niska K, Sund M, Chabok A, Nikberg M, Sigurdadottir J, Schmid R, Werder G, Bluelle R, Frey D, Oswald D, Palma A, Peros G, Reinisch K, Zuk G, Gübeli A, Müller J, Widmer LW, Gerosa A, Mahanty S, Nocito A, Raptis DA, Zuber M, Zumbühl L, Adıyaman C, Bayram S, Cengiz TB, Cevik M, Işler V, Kobal BB, Mutlu D, Ozben V, Ozmen BB, Pektaş AM, Sapci I, Tansoker I, Toto ÖF, Yolcu S, Çakaloğlu HC, Altinel Y, Gulcicek OB, Vartanoglu T, Alis H, Halicioglu I, Sahbaz NA, Arslan E, Baki BE, Bodur S, Celik S, Guner A, Gül E, Murutoglu B, Semiz A, Tomas K, Yildirim R, Aydin MC, Karahan SR, Kose E, Karabulut K, Mutlu V, Ozkan BB, Chen KY, Heard R, Nanthakumaran S, Breslin R, Srinivasan R, Boggon A, Connor K, Haslegrave A, Laurie K, Mann T, Dashnyam E, Kalakouti E, Mehdi A, Post N, Stourton F, Warren O, White R, Paramasivan A, Blencowe N, Bowling K, Bunting D, Ireland P, Reunis E, Soon WC, Tyler R, Kufeji D, Skerritt C, Wright N, Barmayehvar B, Datta U, Kamarajah SK, Karandikar S, Dick L, Liew I, Mairs NG, Qureshi M, Rocke A, Bond-Smith G, Farhangmehr N, Perenyei M, Pezas T, Urbonas T, Alhammali T, Ibrahem AA, Salama Y, Gani MA, Gravante G, Iqbal MR, Jeffery A, Jeon H, Khosla S, Perera J, Jeffery A, Perera J, Kabariti R, Oram S, Chiu S, Cullen F, Kidd T, Owen C, Sarafilovic H, Wilson M, Fouad D, Minocha A, Kadiwar S, Luck J, Smedley A, Currow C, Mykoniatis I, Tani SI, Knight S, Nassif D, Sharma A, Ali W, Dissanayake T, Ho A, Tennakoon A, Lim J, Ng JCK, Gupta A, Shatkar V, Wong F, Donnelly P, Monaghan E, Walker M, Abbas A, Andress C, Bisset C, Chin YR, Evans E, Ishak N, Kamya S, Ploski J, Blackwell J, Herrod P, Lund J, Wakefield R, Keogh K, Longstaff L, Smart N, Ang YL, Camilleri-Brennan J, D'Souza MS, Henshall DE, Lim H, Mclean K, Mirza S, Ng ZH, Park J, Paterson-Brown S, Pronin S, Roy C, Tang L, Teasdale E, Ter EZ, Walls L, Yap S, Cole S, Shrimanker N, Stoddart M, Walker N, Bandi A, Cohen F, Giuliani S, Baillie K, Bamford R, Harvey N, Kershaw S, Nicholson L, Orton P, Palliyil M, Patel S, Shillito S, Abbott T, Akpenyi O, Caydiid H, English W, Hall E, Maciejec L, Mahdi S, Morgan C, Rob Z, Torrance HD, Townsend D, Irwin G, Johnston R, Chowdhury D, Evans D, Patel P, Davies R, Griffiths E, Mansuri A, Nepogodiev D, Jones C, Lim SJ, O'Neill S, Tan C, Dhillon D, Jama GM, Patel K, Al-Bahrani A, Elshaer M, Hunter K, Dindyal S, Majid K, Rajmohan S, Smith C, Chan L, Din F, Eng C, L'Heveder A, McGarvie S, McIntosh K, Park EHG, Ravishankar R, Shahbaz AR, Yau JD, Teasdale E, Blacker S, Kaul A, Parakh J, Awadallah S, Farag S, Nessa A, Beamon M, Caliman C, Duane T, Choudhry A, Haddad N, Zielinski M, Gash K, Kiran RP, Murray A, Narayanan R, Swaroop M, Deal R, Myers J, Schadde E, Hemmila M, Napolitano L, To K, Dasari M, Etchill E, Puyana J, Maimbo M, Makupe A, Musowoya J, Kumwenda D, Otten K, Prins M, Reece-Smith A, Van Der Naald N, Verbeek A, Balmaceda R, Suarez AAB, Deane C, Dijan E, Elfiky M, Koskenvuo L, Buisson P, Henric N, Rod J, Limoges B, Rosello O, Thollot A, Azzis O, Leroux J, Etienne S, Pinnagoda K, Francois P, Alexandre C, Capito C, Hmila S, Kotobi H, Imoro O, Abem OE, Clegg-Lamptey J, Wondoh P, Soulou V, Papageorgiou D, Peña L, Asturias S, Kumar B, O'Connor DB, Taddei A, Ruzzenente A, Notarnicola M, Pascale G, Ubiali P, Luca E De, Sacco M, Pascale MM, Cona C, Rotunno G, Corbellino M, Morandi E, Guglielmo V, Muzio E, NA, Mao P, Bottini C, Luc AR, NA T Bocchetti, Cautiero R, Russo AA, Notarnicola M, Solaini L, Ali FM, Kutkevicius J, Ignatavicius P, Žilinskas J, Baltrunas R, Kondrotas P, Strupas K, Siaw JY, Tan CL, Yam SY, Wilson L, Aziz MRA, Bondin J, Zorrilla CD, Majbar A, Nwabuoku E, Taiwo A, Sale D, Abdullahi L, Faboya O, Fatuga A, Osagie O, Bliksøen M, Khan ZA, Coronel J, Miranda C, Helguero-Santin LM, Vasquez I, Mironescu A, Rickard J, Adedeji A, Alqahtani S, Koto MZ, Rath M, Niekerk M Van, Matos-Puig R, Israelsson L, Schuetz T, Mericliler M, Uluşahin M, Yuksek MA, Farhan-Alanie MMH, Redgrave N, Wilson M, Callan R, Yong GL, Lee K, Wolf B, Musyoka CK, Cox M, Whitehurst K, Fairfield C, Olivier J, Chibuye C

## Abstract

**Background:**

Identification of patients at high risk of surgical-site infections may allow surgeons to minimize associated morbidity. However, there are significant concerns regarding the methodological quality and transportability of models previously developed. The aim of this study was to develop a novel score to predict 30-day surgical-site infection risk after gastrointestinal surgery across a global context and externally validate against existing models.

**Methods:**

This was a secondary analysis of two prospective international cohort studies: GlobalSurg-1 (July–November 2014) and GlobalSurg-2 (January–July 2016). Consecutive adults undergoing gastrointestinal surgery were eligible. Model development was performed using GlobalSurg-2 data, with novel and previous scores externally validated using GlobalSurg-1 data. The primary outcome was 30-day surgical-site infections, with two predictive techniques explored: penalized regression (least absolute shrinkage and selection operator (‘LASSO’)) and machine learning (extreme gradient boosting (‘XGBoost’)). Final model selection was based on prognostic accuracy and clinical utility.

**Results:**

There were 14 019 patients (surgical-site infections = 12.3%) for derivation and 8464 patients (surgical-site infections = 11.4%) for external validation. The LASSO model was selected due to similar discrimination to extreme gradient boosting (AUC 0.738 (95% c.i. 0.725 to 0.750) *versus* 0.737 (95% c.i. 0.709 to 0.765)), but greater explainability. The final score included six variables: country income, ASA grade, diabetes, and operative contamination, approach, and duration. Model performance remained good on external validation (AUC 0.730 (95% c.i. 0.715 to 0.744); calibration intercept −0.098 and slope 1.008) and demonstrated superior performance to the external validation of all previous models.

**Conclusion:**

The ‘Global Surgical-Site Infection’ score allows accurate prediction of the risk of surgical-site infections with six simple variables that are routinely available at the time of surgery across global settings. This can inform the use of intraoperative and postoperative interventions to modify the risk of surgical-site infections and minimize associated harm.

## Teaser

There are significant concerns regarding the methodological quality and transportability of models previously developed to predict patients at risk of surgical-site infections to allow surgeons to minimize associated morbidity. The novel ‘Global Surgical-Site Infection’ score allows accurate prediction of the risk of surgical-site infections with six simple variables that are routinely available at the time of surgery across global settings. This can inform the use of intraoperative and postoperative interventions to modify the risk of surgical-site infections and minimize associated harm.

## Introduction

Surgical-site infections (SSI) are among the most common postoperative complications in patients undergoing gastrointestinal surgery, with the associated postoperative morbidity and mortality posing a significant burden to both patients and health systems^1,2^. There has been a marked reduction in the incidence of SSI as a result of the introduction of numerous perioperative interventions that can reduce the risk and associated harm of SSI^3–5^. Nonetheless, these infections continue to affect 10% of patients in high-income countries and one-third of patients in low- and middle-income countries (LMIC)^6^. To provide an evidence-based approach to allocation of these perioperative interventions, there needs to be better understanding of the patients at greatest risk. Furthermore, the earlier this can be determined in the perioperative care pathway, the more effectively interventions for treatment and surveillance can be provided.

Efforts to develop predictive tools for SSI have been ongoing for decades^7^; however, many do not align with current methodological recommendations^8^ and the few that have been externally validated outside the original cohorts typically display only moderate discrimination^7^. None has been widely adopted in clinical practice^3–5^, reflecting that the feasibility and clinical utility of these models continues to remain uncertain outside of their original contexts. This is particularly important for LMIC, which experience the greatest burden of SSI and stand to benefit most from direction of limited resources towards patients at most risk of SSI^6^. Therefore, the aim of this study was to develop and validate a prognostic score based on data available at the time of operation to predict 30-day SSI risk after gastrointestinal surgery across a global context. Subsequently, the aim was to perform external validation across global data in comparison with existing prognostic models.

## Methods

This study reports the derivation and validation of the ‘Global Surgical-Site Infection’ (GloSSI) risk prediction model to stratify patients by their 30-day SSI risk after major gastrointestinal surgery. This study is reported according to the ‘Transparent reporting of a multivariable prediction model for individual prognosis or diagnosis’ (TRIPOD) statement^9^.

### Data sources

This was a secondary analysis of two independent prospective multicentre international studies conducted by the GlobalSurg Collaborative (www.globalsurg.org). Eligible patients were identified by local collaborators at each participating hospital during 2-week data collection intervals within the respective data collection windows: July–November 2014 (‘GlobalSurg-1’^10^) and January–July 2016 (‘GlobalSurg-2’^11^). These studies collected routine anonymized data with no change to clinical care pathways. Both were registered according to the appropriate local or national approval pathways in each participating country (audit approval, ethical or institutional review board). This secondary analysis was not pre-registered in an independent institutional registry.

Consecutive patients undergoing a broad range of gastrointestinal surgical procedures were eligible. However, each study focused on different cohorts of patients: GlobalSurg-1 focused on those undergoing emergency gastrointestinal surgery for any indication^10^; and GlobalSurg-2 focused on those undergoing emergency and elective gastrointestinal surgery^11^. A full description of methods and primary findings of each of these studies has been previously described^6,12^. Any centres on a global basis that routinely performed these surgeries were eligible to participate in the respective studies. For the purposes of this analysis, only adult patients (greater than or equal to 18 years old, with no upper age limit) undergoing gastrointestinal surgery were included across both studies. This included relevant procedures with no entry of the gastrointestinal tract (for example diagnostic laparotomy/laparoscopy, adhesiolysis, and hernia repair).

### Data collection

Data in both studies were collected using a collaborative research methodology^13^, engaging clinicians, medical students, and allied healthcare professionals at sites around the world to collect data according to pre-published study protocols^10,11^. Anonymized data were submitted and stored on secure research electronic data capture (REDCap) servers^14^. Data collection teams were unaware of the predictors that would be included in the development or external validation of scores and therefore no blinding to outcomes or other predictors was deemed necessary.

Data were collected related to baseline demographics, perioperative care, and 30-day outcomes for each patient using a pre-specified case report form^10,11^. Both data sets were standardized based on variables that shared a common definition and a complementary data structure. Data dictionaries from both studies were extracted directly from the REDCap platform and cross-referenced to identify these variables. These pairings were cross-tabulated before and after combination to ensure consistent order and encoding within the combined data set. These data were further supplemented using country-level World Bank income data for the year of data collection^15^ and BUPA Schedule of Procedures classifications of operative complexity^16^.

### Outcome definition

The primary outcome in this analysis was 30-day SSI. In all GlobalSurg studies, this was defined according to the Centers for Disease Control and Prevention (CDC) definition^17^ (including all superficial, deep, and organ-space infections). All SSI events were recorded at 30 days after surgery either through in-person or health record review. However, in locations where it was not possible to conduct 30-day follow-up due to resource limitations, SSI was measured at the point of discharge, with a pragmatic assumption that SSI would not occur post-discharge. This CDC definition of SSI was used for all external validation models, irrespective of the exact definition used in the original derivation models.

### Model derivation

The GlobalSurg-2 data set was selected as the derivation cohort due to having the broadest inclusion criteria and the primary outcome being 30-day SSI^11^. The minimum sample size required for developing a multivariable prediction model was explored across a range of plausible scenarios based on the number of parameters, event rates, and expected model discrimination^18^ (*Fig. S1*). Based on the event rate previously reported for the GlobalSurg-2 data set^6^, the sample size was anticipated to be adequate for a model with an area under the curve (AUC) greater than or equal to 0.65. Candidate variables were considered from those previously included in prognostic models identified in a systematic review^7^. Additional variables were considered based on potential relevance and appropriateness to resource-limited environments. Candidate variables were discarded if: there was no clear clinical rationale for an influence on the outcome; they were not generalizable to all patients undergoing gastrointestinal surgery; or they would not be expected to be accessible across income settings. A priori, it was decided that any missing data would be handled using multiple imputation with chained equations, under the ‘missing-at-random’ assumption^19^. A total of ten data sets were imputed using available explanatory variables and the outcome variable, with patients clustered by country. Modelling was performed on imputed data sets, with Rubin’s rules used to combine results^20^.

**Fig. 1:**
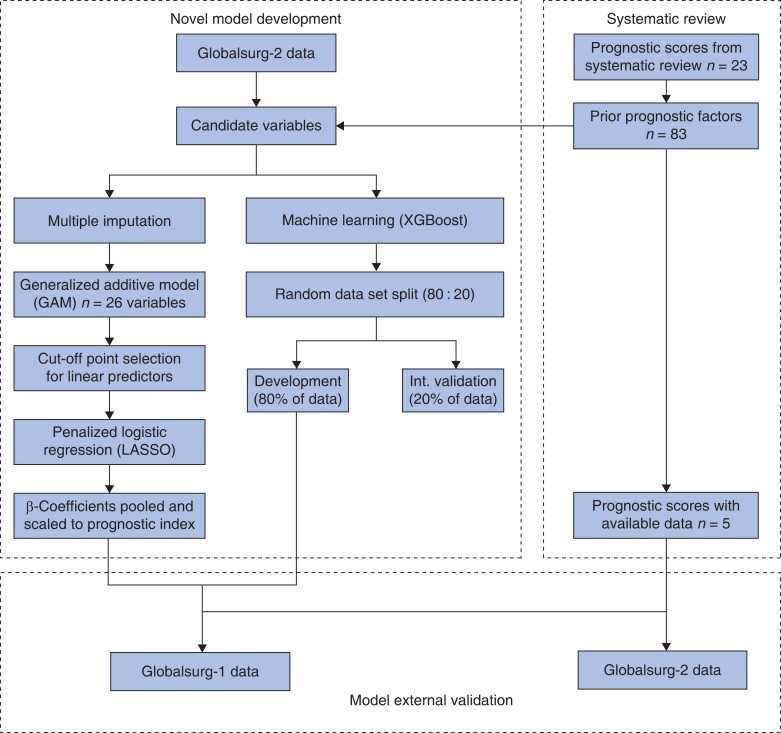
Flow chart of modelling process XGBoost, extreme gradient boosting; Int., Internal; LASSO, least absolute shrinkage and selection operator.

A four-stage model building process was used, in line with best practice^21^ (*Fig. 1*). First, a criterion-based approach with a generalized additive model was used for initial variable selection^22^. Each variable was omitted from the complete model in turn (‘leave-one-out’ approach), with the decision to retain a variable based on the change in either the deviance explained (greater than 1%) or unbiased risk estimator (greater than 10%) compared with the complete model. Second, any continuous variables were categorized to facilitate clinical utility based on the observed relationship with SSI in the generalized additive model using an approach previously shown to maintain discriminative performance^22^. Third, a least absolute shrinkage and selection operator (LASSO) logistic regression model was used to establish the simplest model with the highest predictive potential. The final variables included were selected based on being retained in the majority (greater than five) of imputed data sets. Fourth, these selected variables were entered into a mixed-effects logistic regression model, with patients clustered by country. The fixed-effects coefficients across the models were pooled using Rubin’s rules^23^ and scaled to create the GloSSI prognostic index. As disparities in outcomes at country income level are due to systematic country differences, rather than being relevant at an individual level, these differences were instead accounted for by model intercepts specific for income level.

To provide a comparison of the predictive potential of the available data by accounting for any complex underlying interactions, an alternative machine-learning approach was explored (extreme gradient boosting (XGBoost))^21^. This included all candidate predictor variables from the development data set that were present within the external validation data set. The development data set was randomly split into training and testing sets in an 80 : 20 ratio, with models fitted through ten-fold cross-validation, minimizing the binary classification error rate (the proportion of errors in classification of patients with SSI). Hyperparameters were also tuned via grid search to maximize the AUC in the test set.

### External validation

External validation of the final GloSSI model was performed using the GlobalSurg-1 data set^12^. Furthermore, all previous prognostic models identified in a systematic review were externally validated when the corresponding clinical parameters were available in the GlobalSurg data sets^7^. Where appropriate, clinical parameters that were aligned, but not exactly equivalent, in the GlobalSurg data sets were rationalized on a pragmatic basis. Operative duration is a key variable used in previous prognostic models and was collected in GlobalSurg-2, but not GlobalSurg-1. Therefore, a separate multivariable linear regression model was developed using GlobalSurg-2 data and used to estimate operative duration in the validation data set based on all patient and operative characteristics common across both data sets (*Table S1*). Missing data were handled in the validation data set as per the derivation cohort, except the outcome variable was excluded as a predictor.

### Statistical analysis

Model performance was compared using the AUC and prognostic accuracy summary statistics calculated for a range of cut-off values (sensitivity, specificity, positive predictive value (PPV), and negative predictive value (NPV)). An AUC with a lower confidence interval of 0.5–0.59 was considered to indicate ‘poor’ model discrimination, 0.6–0.69 was considered to indicate ‘moderate’ model discrimination, 0.7–0.79 was considered to indicate ‘good’ model discrimination, and greater than or equal to 0.8 was considered to indicate ‘excellent’ model discrimination^24^. Calibration was assessed through visual inspection and the calibration intercept (calibration-in-the-large) and slope^25^ (an intercept of 0 and slope of 1 indicates ‘perfect’ calibration). No recalibration was planned or performed to allow determination of whether these models were ‘transportable’ in their original iteration^26^ (for example continue to produce accurate predictions in a related, but different, population). Finally, a decision curve analysis was performed, which can allow determination of the clinical utility of a prognostic model through comparison of the relative value of benefits (treating a true positive) and harms (treating a false positive)^27^. All statistical analyses were performed in R Studio version 4.1.1 (R Foundation for Statistical Computing, Vienna, Austria), with packages including tidyverse, finalfit, and predictr^28^.

## Results

### Cohort characteristics

Data for 22 483 patients were eligible to be included in modelling (*Fig. 1*): 14 019 patients (62.4%) in the derivation cohort (GlobalSurg-2) and 8464 patients (37.6%) in the validation cohort (GlobalSurg-1).

Within the development cohort, the overall SSI rate was 12.3% (1730 patients), including 488 patients (3.5%) with organ-space infections (*Table 1*). The median age of patients in the cohort was 47.0 (interquartile range 32.0–63.0) years; 7051 patients (50.3%) were female and 8089 patients (57.7%) had at least one co-morbidity (ASA grade II–V). Half of the development cohort (6877 patients; 49.1%) were from LMIC. However, there were substantial differences in sociodemographic and clinical characteristics between the surgical populations in the derivation cohort and the validation cohort (*Table 1*). GlobalSurg-1 only included patients requiring emergency procedures, who were substantially more co-morbid and undergoing procedures that were technically less complex, yet often more contaminated and performed using an open approach. The overall SSI rate observed was broadly consistent between the cohorts (12.3% (1730 of 14 019) in the derivation cohort *versus* 11.4% (965 of 8464) in the validation cohort).

**Table 1:** Characteristics of the development and external validation cohorts

Characteristic	Derivation cohort (GlobalSurg-2)	Validation cohort (GlobalSurg-1)	*P*
**Age (years), mean(s.d.)**	47.9(18.6)	46.1(20.3)	<0.001
**Sex**			
Male	6184 (44.1)	4461 (52.7)	<0.001
Female	7051 (50.3)	4000 (47.3)	
Missing	784 (5.6)	3 (0.0)	
**History of smoking**			
Non-smoker	8538 (60.9)	4883 (57.7)	<0.001
Ex-smoker	1643 (11.7)	978 (11.6)	
Current smoker	2248 (16.0)	1616 (19.1)	
Missing	1590 (11.3)	987 (11.7)	
**History of diabetes**			
No	12 653 (90.3)	7495 (88.6)	<0.001
Diet controlled	120 (0.9)	166 (2.0)	
Medication (non-insulin) controlled	801 (5.7)	371 (4.4)	
Insulin controlled	376 (2.7)	210 (2.5)	
Missing	69 (0.5)	222 (2.6)	
**ASA grade**			
I	5544 (39.5)	3438 (40.6)	<0.001
II	5486 (39.1)	2692 (31.8)	
III	2162 (15.4)	1418 (16.8)	
V	94 (0.7)	178 (2.1)	
Missing	386 (2.8)	264 (3.1)	
**History of immunosuppressive medication**				
No	13 739 (98.0)	–	–
Yes	195 (1.4)	–	
Missing	85 (0.6)	–	
**History of HIV**			
No	12 701 (90.6)	–	–
Yes	83 (0.6)	–	
Missing	1235 (8.8)	–	
**History of corticosteroid use**			
No	13 571 (96.8)	–	–
Yes	333 (2.4)	–	
Missing	115 (0.8)	–	
**History of chemotherapy (<6 weeks)**			
No	13 646 (97.3)	–	–
Yes	293 (2.1)	–	
Missing	80 (0.6)	–	
**Preoperative antibiotic treatment**			
No	10 383 (74.1)	–	–
Yes	3385 (24.1)	–	
Missing	251 (1.8)	–	
**Operative pathology**			
Benign	11 233 (80.1)	7460 (88.1)	<0.001
Trauma	259 (1.8)	488 (5.8)	
Malignant	2523 (18.0)	516 (6.1)	
Missing	4 (0.0)	0 (0.0)	
**Operative contamination**			
Clean-contaminated	11 032 (78.7)	6907 (81.6)	<0.001
Contaminated-dirty	2835 (20.2)	1557 (18.4)	
Missing	152 (1.1)	0 (0.0)	
**Intraoperative perforation**			
No	13 617 (97.1)	6879 (81.3)	<0.001
Yes	402 (2.9)	1557 (18.4)	
**Operative urgency**			
Elective	7653 (54.6)	0 (0.0)	<0.001
Emergency	6366 (45.4)	8464 (100.0)	
**Operative duration (min), mean(s.d.)**			
	117.4(88.1)	–	–
**Operative speciality**			
UGI	2182 (15.6)	1150 (13.6)	<0.001
HPB	5074 (36.2)	1211 (14.3)	
Colorectal	5663 (40.4)	4291 (50.7)	
Other*	1100 (7.8)	1812 (21.4)	
**Intraoperative stoma formation**			
No	13 647 (97.3)	7592 (89.7)	<0.001
Ileostomy	135 (1.0)	359 (4.2)	
Colostomy	237 (1.7)	441 (5.2)	
Other	0 (0.0)	65 (0.8)	
**Operative complexity**			
Intermediate	2528 (18.0)	2983 (35.2)	<0.001
Major	7468 (53.3)	3554 (42.0)	
Major+	2614 (18.6)	1159 (13.7)	
Complex major	1393 (9.9)	762 (9.0)	
Missing	16 (0.1)	6 (0.1)	
**Operative approach**			
Minimally invasive	6960 (49.6)	2791 (33.0)	<0.001
Open	7059 (50.4)	5668 (67.0)	
**Country World Bank income**			
Low/lower-middle	4072 (29.0)	1928 (22.8)	<0.001
Upper-middle	2805 (20.0)	1254 (14.8)	
High	7142 (50.9)	5282 (62.4)	
**30-day SSI rate**			
No	11 545 (82.4)	7452 (88.0)	0.001
Yes	1730 (12.3)	965 (11.4)	
Missing	744 (5.3)	47 (0.6)	

Values are *n* (%) unless otherwise indicated. *Procedures with no entry of the gastrointestinal tract. HIV, human immunodeficiency virus; UGI, Upper gastrointestinal; HPB, Hepato-Pancreato-Biliary; SSI, surgical-site infection.

### Development of a novel model

A total of 23 candidate predictor variables were identified within the development data set that were assessed before surgery or intraoperatively (*Table S2*). A further four variables were derived from existing variables (operative duration and specialty) or linked from external data sets (World Bank income level and operative complexity (BUPA Schedule of Procedures)). Data on these candidate variables were complete for 9762 patients (69.6%) and the pattern of missing data was consistent with a ‘missing-at-random’ assumption (*Fig. S2a*). Due to small numbers within the development data set, co-morbidities describing the characteristic of immunosuppression (human immunodeficiency virus or malarial infection, corticosteroid or other immunosuppressant use, and recent chemotherapy) were collapsed into a single binary variable.

Generalized additive models were applied to the imputed data sets then pooled, with eight meeting the a priori threshold to be considered as important predictors of SSI (*Table S3*). Component smoothed functions generated were used to select informative cut-off values to categorize continuous variables (age and operative duration) (*Fig. S3*). On entering these variables into a penalized logistic regression model (LASSO), all variables were retained within the final model. Penalized regression coefficients of the final model (*Table 2*) were scaled into a prognostic index (GloSSI score: range 0–74).

**Table 2:** ‘Global Surgical-Site Infection’ score for prediction of surgical-site infections in adults undergoing gastrointestinal surgery

Variable		Level	β-Coefficient	Points
**Co-morbidities**	ASA grade	I	0	0
		II	0.245	5
		III	0.517	10
		IV	0.633	13
		V	0.291	6
	History of diabetes	No	0	0
		Diet controlled	0.591	12
		Medication (non-insulin) controlled	0.334	7
		Insulin controlled	0.354	7
**Operative**	Operative approach/contamination	Minimally invasive/clean-contaminated	0	0
		Open/clean-contaminated	1.014	20
		Minimally invasive/contaminated-dirty	0.422	8
		Open/contaminated-dirty	1.968	39
	Operative duration (min)	<200	0.000	0
		≥200	0.501	10

Model intercept by country income level: high income = −70 (β = −3.49), upper middle income = −63 (β = −3.15), and low and lower middle income = −60 (β = −2.98). To generate the final scaled score, mean coefficients were multiplied by 20 and rounded.

**Fig. 2:**
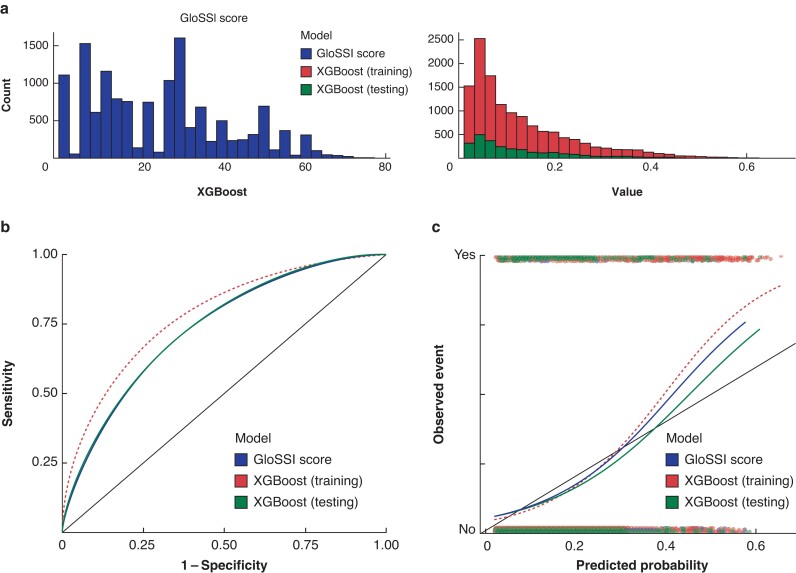
Comparison of the performance of the least absolute shrinkage and selection operator and the extreme gradient boosting modelling approaches in the development cohort (GlobalSurg-2) **a** Model prediction distribution. **b** Receiver operating characteristic (ROC) curves. **c** Calibration curves. GloSSI, ‘Global Surgical-Site Infection’; XGBoost, extreme gradient boosting.

The pooled LASSO model demonstrated good discrimination (AUC 0.738 (95% c.i. 0.725 to 0.750)) and calibration (intercept 0.044 and slope 0.995) for 30-day SSI within the derivation cohort (*Table 3* and *Fig. 2*). This was similar to the XGBoost model within the internal validation (test) data set, which also showed good discrimination (AUC 0.737 (95% c.i. 0.709 to 0.765)) and high calibration (intercept −0.046 and slope 0.995) across the range of risk (*Fig. 2*). However, both models underestimated the probability in those at highest risk of SSI (*Table 3* and *Fig. 2b*). Using the GloSSI prognostic index (*Table 2* and *Table S4*), the prognostic accuracy was investigated across a range of values to provide flexibility in adapting to different clinical use cases (*Table 4*). With a ‘rule in’ approach, to exclude ‘low-risk’ patients (GloSSI score less than or equal to 5), a NPV of 95.9% (sensitivity 89.9% and specificity 33.5%) can be achieved. In contrast, with a ‘rule out’ approach, to select only ‘high-risk’ patients (GloSSI score greater than 50), an PPV of 36.2% (sensitivity 12.9% and specificity 96.8%) can be achieved.

**Table 3:** Model performance of the ‘Global Surgical-Site Infection’ score and previous predictive models across the GlobalSurg data sets

Model	Status	GlobalSurg-1		GlobalSurg-2	
		AUC (95% c.i.)	Calibration intercept and slope	AUC (95% c.i.)	Calibration intercept and slope
**GloSSI (LASSO)**	Derivation	–	–	0.738 (0.725,0.750)	0.044 and 0.995
	External validation	0.730 (0.715,0.744)	−0.098 and 1.008	–	–
**XGBoost**	Derivation	–	–	0.783 (0.770,0.796)	−0.002 and 1.192
	Internal validation	–	–	0.737 (0.709,0.765)	−0.046 and 0.940
	External validation	0.728 (0.713,0.743)	−0.298 and 0.926	–	–
**Grant *et al*.^41^ (2019)**	External validation	0.696 (0.680,0.711)	–	0.696 (0.683,0.710)	–
**NNIS (1986)**	External validation	0.617 (0.600,0.635)	0.789 and 1.007	0.627 (0.614,0.640)	0.923 and 1.175
**RSSIC (2012)**	External validation	–	–	0.605 (0.592,0.618)	–
**SENIC (1985)**	External validation	0.635 (0.617,0.653)	0.362 and 0.688	0.659 (0.646,0.673)	0.461 and 0.882
**Updated NNIS (2001)**	External validation	0.687 (0.673,0.702)	0.969 and 1.445	0.698 (0.686,0.711)	1.203 and 1.308

GloSSI, ‘Global Surgical-Site Infection’; LASSO, least absolute shrinkage and selection operator; XGBoost, extreme gradient boosting; NNIS, National nosocomial infections surveillance system; RSSIC, Risk of Surgical Site Infection in Cancer; SENIC, Study on the Efficacy of Nosocomial Infection Control.

**Table 4:** ‘Global Surgical-Site Infection’ score cut-off values and prognostic accuracy using the derivation data set

Cut-off value	Surgical-site infection rate (%)		Prognostic accuracy using the GlobalSurg-2 data set			
	‘Low’ risk (<cut-off)	‘High’ risk (≥cut-off)	Sensitivity (%)	Specificity (%)	PPV (%)	NPV (%)
**5**	4.1 (*n* = 175/4282)	16.1 (*n* = 1566/9737)	89.9	33.5	16.1	95.9
**10**	4.5 (*n* = 240/5384)	17.4 (*n* = 1501/8635)	86.2	41.9	17.4	95.5
**15**	4.9 (*n* = 308/6323)	18.6 (*n* = 1433/7696)	82.3	49.0	18.6	95.1
**20**	6.4 (*n* = 547/8532)	21.8 (*n* = 1194/5487)	68.6	65.0	21.8	93.6
**30**	7.7 (*n* = 820/10 659)	27.4 (*n* = 921/3360)	52.9	80.1	27.4	92.3
**40**	9.6 (*n* = 1182/12 336)	33.2 (*n* = 559/1683)	32.1	90.8	33.2	90.4
**50**	11.3 (*n* = 1516/13 398)	36.2 (*n* = 225/621)	12.9	96.8	36.2	88.7

PPV, positive predictive value; NPV, negative predictive value.

### External validation of the novel model and comparison with previous predictive models

There were 23 previous models identified in a systematic review^7^. All common model parameters (identified in greater than or equal to three models) were present in one or more GlobalSurg data set (*Fig. S4*), apart from BMI (n=10/23). Overall, five prior models were able to be externally validated using one or both of the GlobalSurg data sets. Of the 18 models that could not be validated due to a lack of prerequisite clinical parameters being present, this was predominantly due to an absence of preoperative blood test results or other infrequently identified predictors not being present (*Fig. S4*). Only four additional models could have been externally validated if BMI had been present in these data sets^29–32^.

There were substantial differences between the surgical populations in the GlobalSurg cohorts and cohorts used to derive the previous scores undergoing external validation, with comparisons limited by the data reported (*Tables S5*, *S6*). Most notably, 40% (two of five) of previous scores were developed using data from patients undergoing non-abdominal procedures, with up to 70% of procedures reported to be classified as clean surgery. Furthermore, all previous scores were developed in cohorts from upper-middle- or high-income countries only.

**Fig. 3:**
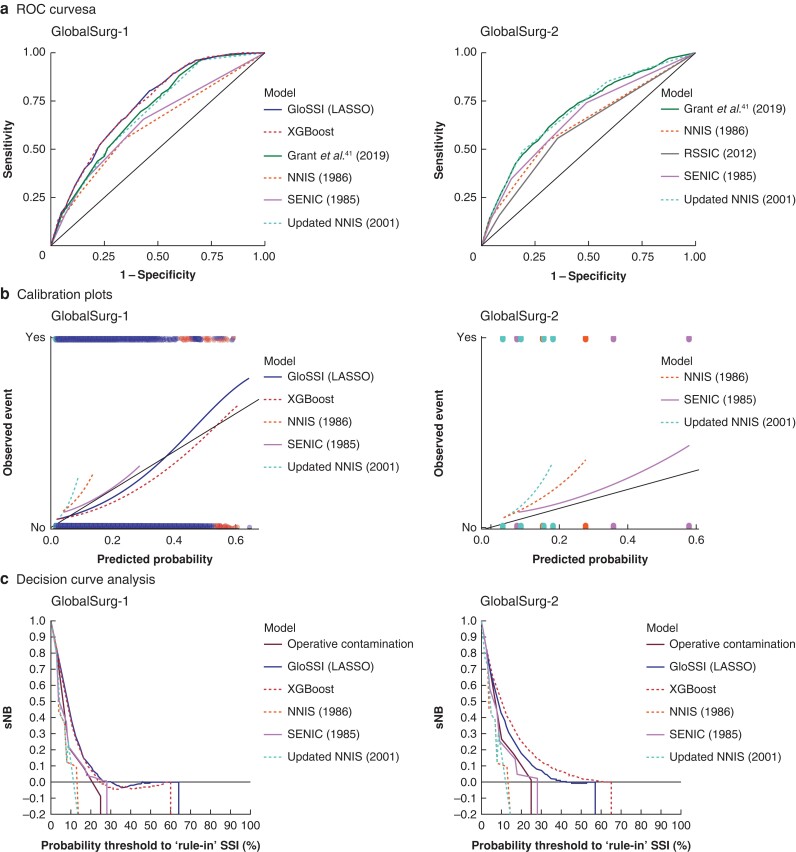
External validation of the ‘Global Surgical-Site Infection’ score and previous predictive models across the GlobalSurg data sets ROC, receiver operating characteristic; GloSSI, ‘Global Surgical-Site Infection’; LASSO, least absolute shrinkage and selection operator; XGBoost, extreme gradient boosting; NNIS, National nosocomial infections surveillance system; SENIC, Study on the Efficacy of Nosocomial Infection Control; RSSIC, Risk of Surgical Site Infection in Cancer; sNB, standardised net benefit.

On external validation using the GlobalSurg data sets, all models demonstrated a consistent performance across both data sets (*Table 3* and *Fig. S5a*). The GloSSI score and XGBoost comparator demonstrated consistently superior discrimination across the validation data set compared with previous models (*Table 3*, *Fig. 3a*, and *Fig. S5a*) and remained statistically significant ‘good’ discrimination for SSI. Sensitivity analysis to explore the results of external validation using only LMIC data showed that the GloSSI score and XGBoost comparator performed best among the scores evaluated, although with a reduction in discrimination compared with the full data sets (*Fig. S5b*).

Nevertheless, the GloSSI score and XGBoost comparator appeared to remain well calibrated across a wide spectrum of risk on external validation (*Table 3* and *Fig. 3b*). For the three models with sufficient information to determine calibration, these had a narrower spectrum of predicted probabilities with typically poor calibration demonstrated across the GlobalSurg data sets (*Table 3* and *Fig. 3b*). Subsequent decision curve analysis showed that the GloSSI and XGBoost models had better clinical utility across a wider range of threshold risks compared with prior prognostic scores for the GlobalSurg data sets (*Fig. 3c*).

## Discussion

This analysis used robust and well-established modelling techniques to derive a novel prognostic model (GloSSI score) to predict the likelihood of SSI within the first 30 days after surgery in those undergoing gastrointestinal surgery. This was developed using prospective data from an observational study conducted on a global basis and incorporates predictor variables that are accessible to clinicians across all settings and at the time of surgery. On external validation in an emergency surgery population, the GloSSI score was demonstrated to be well calibrated across a wide spectrum of risk, with superior discrimination and clinical utility on decision curve analysis when compared with existing prognostic models. Furthermore, of the five prognostic models able to be externally validated using the large prospective GlobalSurg data sets, none was significantly higher than the a priori threshold for ‘good’ discrimination in the cohort (AUC greater than or equal to 0.7) or consistently acceptable calibration.

Prediction of the risk of SSI at the point of surgery remains a challenging task, with a breadth of prognostic factors previously identified^7^. However, methodological issues in previous models have made it difficult to determine the true importance of these factors in the development of SSI. The variable selection process for the GloSSI score prioritized several of the co-morbidities and operative factors most commonly identified in previous models (*Fig. S4*), emphasizing their importance in a global context. However, the combination of variables and weightings in the GloSSI score remains distinct from all pre-existing scores developed. Furthermore, these were a limited number of simple variables that are routinely available across income settings, increasing the clinical utility within routine practice. It was also notable that the effects of factors commonly highlighted in past models (for example patient age, history of smoking, and operative urgency) were largely explained by the contributions of other factors within the model. While this does not exclude an effect from these variables, it suggests that the factors included in the GloSSI model have a far greater value in the prognosis of SSI. Country income level was also identified as a significant prognostic factor for SSI (*Table 2*). This is a crude surrogate for a multitude of potentially causal factors, including differences in surgical practice, access to surgical care, presentation of pathologies, and availability of resources^33–35^. This may account for the lower performance on external validation in the LMIC subgroups, despite the incorporation of this factor into the model (*Fig. S5*).

The GloSSI score demonstrated consistently superior discrimination and clinical utility on decision curve analysis across the external validation data set compared with previous models, which were typically aligned with those reported in previous validation studies^7^ (*Fig. S5*). However, regarding the previous scores for which calibration was able to be evaluated (*Fig. 3b*), poor calibration was generally observed. This may be in part due to an earlier ceiling to the predicted probabilities compared with the GloSSI score, which demonstrated good calibration across a range of risks of SSI. This indicates that the GloSSI score is suitable for the reliable prediction of SSI in a broad cohort of undifferentiated patients undergoing gastrointestinal surgery, particularly those undergoing emergency procedures. However, further external validation using other data sets, particularly elective surgery populations, would be beneficial.

In recognition of the theoretical benefits of machine-learning approaches for clinical prediction^36^, an XGBoost model was also developed as a best-in-class alternative of the primary modelling approach used in this paper. While this demonstrated superior performance within the development cohort, there was equivalent performance to the GloSSI model on external validation (AUC ∼0.73) (*Table 3*). This indicates an element of overfitting in the original data set, but also potentially a limit to the capabilities of prediction of SSI using data that are routinely available at the time of surgery. While further improvement in the predictive potential via machine-learning approaches may be achievable, there is an inherent trade-off in the clinical utility. For example, including a greater number and complexity of variables would increase the burden and barriers to completion, integrating data on the postoperative journey of patients may prevent early intervention, and reduced transparency in the prediction process may reduce explicability and so the willingness of clinicians and patients to use^37^.

This work presents the most comprehensive analysis to date of risk scoring systems for the prediction of SSI after gastrointestinal surgery, with several notable advantages to the approach used to develop and validate the GloSSI score. First, the data sets used throughout these analyses are from among the largest prospective studies of postoperative outcomes and were conducted on a global basis across a spectrum of income settings. This facilitated generalizability of the work to include LMIC settings, which have been overlooked in this area, yet have the highest burden of disease^6,7^. Furthermore, the data collected across the GlobalSurg studies represent almost all common prognostic factors available at the time of surgery previously identified (*Fig. S4*) and so maximized the likelihood of developing an accurate prognostic model. Second, the methodology to develop the GloSSI score was consistent with current best practice. It was determined a priori that there was suitable statistical power for model development, with the low rates of missing data in the GlobalSurg data sets being handled via multiple imputation to maintain this. Candidate variables considered were those with a clear potential role in the causal pathway for the development of SSI and only those available at the time of surgery were considered, increasing the applicability to clinical care. Robust modelling approaches for variable selection were subsequently utilized (including penalized regression and machine-learning approaches), avoiding the issues in using statistical significance as the decision criteria and accounting for collinearity in variables considered^8^. Finally, the largest external validation of prognostic models for SSI in gastrointestinal surgery to date was conducted, including the majority of scores previously externally validated^32,38–40^, as well as two additional scores^41,42^. This allowed a direct and fair comparison of their respective performance alongside the GloSSI score for broad cohorts of patients undergoing gastrointestinal surgery, allowing determination of the improved performance.

However, there are also several important limitations to this analysis. First, not all variables identified as predictors in previous scores were collected within the GlobalSurg studies, for example BMI (*Fig. S4*). This was a pragmatic decision to ensure these studies could be feasibly delivered across a range of income settings, where additional tests or equipment required for collecting data for other variables may not be available. Within this analysis, this limited the data available for score development and to conduct external validation, with the majority of previous scores (17 of 23) remaining unvalidated^7^. However, these unvalidated models had other methodological or practical challenges that limited their suitability in this context of undifferentiated patients undergoing gastrointestinal surgery^7^. This is reflective of broader issues with reproducibility across the prediction literature^43^. Second, the discrimination observed on external validation of models may have been influenced by pragmatic decisions to derive additional variables that were essential to the model. Operative duration was frequently identified as a key prognostic factor across previous models (*Fig. S4*) and was an important feature in the context of the development of the GloSSI score (*Table 2*). However, this variable was only collected for the derivation cohort (GlobalSurg-2) and not the validation cohort (GlobalSurg-1). In the absence of alternative public sources of data on operative duration that were relevant in a global context, data from GlobalSurg-2 were used to predict operative duration in the validation data set (*Table S1*). While this may theoretically reduce discrimination for previous models, all scores bar one (Grant *et al*.^41^) categorize operative duration into broad intervals and so would not explain the typically poor discrimination observed. In contrast, there is a risk of information leakage from the derivation cohort to the validation cohort, given that the operative duration was predicted using variables shared with the GloSSI model. As such, it is possible that the performance of the GloSSI model in the validation cohort was overestimated and further external validation would be needed to explore this. Third, several pragmatic decisions were made regarding variables in the GlobalSurg data set to facilitate the modelling process. Where exposure rates were low for individual predictors in the GlobalSurg studies, but a common mechanism was shared, these were combined into a composite variable. In the case of ‘history of immunosuppression’, these included variables involving different immunosuppressive mechanisms and so may have different individual contributions to SSI that therefore were not observed. However, none of these variables has been observed to have a strong association with SSI in prior prognostic models^7^ and so would be expected to require significantly larger data sets to determine whether a statistically significant association was present. Similarly, where the data collected in the GlobalSurg data sets did not exactly match the original study, variables were equated where appropriate (*Tables S5*, *S6*). Therefore, this may have also contributed to reduced discrimination observed on external validation for these models. Fourth, while inclusion of World Bank income status was identified as an important predictor of SSI, this is a surrogate measure that is reflective of potential differences in access, pathology, and clinical practice between regions rather than being reflective of intrinsic risk. Furthermore, there can also be substantial variation between countries and between hospitals within countries regarding these factors^44^, which may influence the risk of SSI. Therefore, while differences between World Bank income status are accounted for within the GloSSI score, emphasis is placed on the patient-level factors for clinical decision-making (*Table 2*). Finally, the GlobalSurg studies used the same standard CDC diagnostic criteria for SSI; however, in global settings where it was not possible to conduct outpatient follow-up, the outcome was determined at the point of discharge. Increasingly, SSI has become a complication of the post-discharge interval^45^ and so the true 30-day SSI rate may have been underestimated. This may explain the reduced discrimination in SSI recorded post-discharge within LMICs (*Fig. S5*). Therefore, while the SSI recorded here represents only those that came to the attention of hospitals, this is nonetheless reflective of clinical practice, particularly for areas of poor community healthcare access.

The GloSSI score allowed accurate prediction of the risk of SSI across an undifferentiated cohort of patients undergoing gastrointestinal surgery. It used six simple variables that are routinely available at the time of surgery across global settings and demonstrated superior performance on external validation in an emergency surgery population to all previous models evaluated to date and equivalent performance to a machine-learning-based comparator. It has a clear clinical application to inform intraoperative and postoperative interventions that can modify the risk of SSI and minimize associated harm^3–5^. Without a substantial change in the type and/or modality of data collected during hospital admission, it is unlikely that high discrimination can be achieved using data routinely available at the time of operation. Therefore, if a prognostic score is to be used in clinical care, there must be a pragmatic trade-off between sensitivity and specificity depending on the specific intervention and clinical context being considered. For interventions such as antibiotic prophylaxis, a low sensitivity may be acceptable so long as there remains a high NPV. However, for interventions that are resource-intensive (for example enrolment in clinical trials or enhanced postoperative surveillance programmes), a higher sensitivity may be prioritized to ensure an evidence-based approach to direct these towards patients at highest risk^3,46^. Score cut-offs to facilitate the use of the GloSSI score for these different clinical purposes are provided (*Table 4*). Nonetheless, it should be noted that there has been increasing interest in the use of a dynamic risk model for prognosis in other clinical contexts^47^. Unlike traditional modelling methods, the likelihood of SSI could instead be updated at pre-specified time points (for example before surgery, intraoperatively, and on discharge) to guide evidence-based decision-making throughout the clinical pathway. As surgical demand continues to grow in light of efforts to provide universal healthcare coverage and to address the post-pandemic elective surgical backlog^48,49^, the burden posed by SSI to health systems is expected to continue to scale accordingly. Therefore, there becomes an even greater clinical need for the use of prognostic tools to allow the modifiable risk of SSI to be mitigated. However, while there are clear cases of clinical use for numerous predictive models published, model performance is not a guarantee of clinical adoption^50^. Further studies on the clinical impact or on how clinical prediction tools can be sustainably implemented within care pathways are warranted, rather than reliance on gradual diffusion and uptake into clinical practice.

## Collaborators

**NIHR Global Research Health Unit on Global Surgery and GlobalSurg Collaborative.**

**Writing group**: KA McLean, SR Knight, N Clark, A Ademuyiwa, A Adisa, M Aguilera-Arevalo, D Ghosh, PD Haque, I Lawani, A Ramos-De la Medina, F Ntirenganya, S Samuel, S Tabiri, JF Simões, CA Shaw, SK Kamarajah, M Picciochi, R Pius, T Pinkney, E Li, D Morton, D Nepogodiev, JC Glasbey, A Bhangu, EM Harrison.

**Protocol development**: AO Ademuyiwa, AO Adisa, ML Aguilera, A Altamini, P Alexander, SW Al-Saqqa, G Borda-Luque, J Cornick, A Costas-Chavarri, TM Drake, SJ Fergusson, JE Fitzgerald, J Glasbey, JA Ingabire, L Ismaïl, Z Jaffry, HK Salem, C Khatri, A Kirby, ATT Kojo, MC Lapitan, R Lilford, AL Mihaljevic, M Mohan, D Morton, AZ Mutabazi, D Nepogodiev, F Ntirenganya, R Ots, F Pata, T Pinkney, T Poškus, AU Qureshi, A Ramos-De la Medina, S Rayne, G Recinos, K Søreide, CA Shaw, S Shu, R Spence, N Smart, S Tabiri, EM Harrison, A Bhangu

**Patient representatives**: A Verjee, E Runigamugabo

**Protocol Translators**: THA Ali, S Rekhis, M Rommaneh, O Halhouli (Arabic); ZH Sam (Chinese); L Ismaïl (French); V Kalles (Greek); F Pata, GE Nita, F Coccolini, L Ansaloni (Italian); TB Pugliesi (Portuguese); R Blanco (Spanish)

**GlobalSurg-1 National Leads**: N Gobin (Australia); AV Freitas (Brazil); N Hall (Canada); S Kim (China & Hong Kong); A Negida, H Khairy (Egypt); Z Jaffry, SJ Chapman (England); AP Arnaud (France); S Tabiri (Ghana); G Recinos (Guatemala); Cutting Edge Manipal, M Mohan (India); R Amandito (Indonesia); M Shawki (Iraq); M Hanrahan (Ireland); F Pata (Italy); C Khatri (Lead coordinator); J Zilinskas (Lithuania); AC Roslani, CC Goh (Malaysia); AO Ademuyiwa (Nigeria); G Irwin (Northern Ireland); S Shu, L Luque (Peru); H Shiwani, A Altamimi (Saudi Arabia); SJ Fergusson (Scotland); R Spence, S Rayne (South Africa); J Jeyakumar (Sri Lanka); Y Cengiz (Sweden); DA Raptis (Switzerland); JC Glasbey (Wales)

**GlobalSurg-2 National Leads**: MM Modolo (Argentina); D Iyer, S King, T Arthur (Australia); SN Nahar (Bangladesh); A Waterman (Barbados); L Ismaïl (Benin); M Walsh (Botswana); A Agarwal, A Zani, M Firdouse, T Rouse (Canada); Q Liu (China); JC Correa (Colombia); HK Salem (Egypt); P Talving (Estonia); M Worku (Ethiopia); A Arnaud (France); S Tabiri (Ghana); V Kalles (Greece); ML Aguilera, G Recinos (Guatemala); B Kumar, S Kumar (India); R Amandito (Indonesia); R Quek (Ireland); F Pata, L Ansaloni (Italy); A Altibi (Jordan); D Venskutonis, J Zilinskas, T Poskus (Lithuania); J Whitaker (Madagascar); V Msosa (Malawi); YY Tew (Malaysia); A Farrugia, E Borg (Malta); A Ramos-De la Medina (Mexico); Z Bentounsi (Morocco); AO Ademuyiwa (Nigeria); K Søreide (Norway); T Gala (Pakistan); I Al-Slaibi, H Tahboub, OH Alser (Palestinian Territory); D Romani, S Shu (Peru); P Major (Poland); A Mironescu, M Bratu, A Kourdouli (Romania); A Ndajiwo (Saint Kitts and Nevis); A Altwijri, MU Alsaggaf, A Gudal, JJ Al-Faifi (Saudi Arabia); S Seisay (Sierra Leone); B Lieske (Singapore); S Rayne, R Spence (South Africa); I Ortega (Spain); J Jeyakumar, KJ Senanayake (Sri Lanka); O Abdulbagi (Sudan); Y Cengiz (Sweden); D Raptis (Switzerland); Y Altinel (Turkey); C Kong, E Teasdale, G Irwin, M Stoddart, R Kabariti, S Suresh (United Kingdom); K Gash, R Narayanan (United States); M Maimbo (Zambia)

**GlobalSurg-1 Local Collaborators**: R Balmaceda, C Fermani, MM Modolo (Hospital Luis Lagomaggiore, Argentina); R Chenn, M Edye, N Gobin, E Macdermid, CO Yong (Blacktown Hospital, Australia); SK D'amours, D Iyer, M Jarmin (Liverpool Hospital, The University Of New South Wales, Australia); J Brown, N Phillips, D Youssef (Royal Brisbane & Women's Hospital, Australia); R George, C Koh, O Warren (The Royal Prince Alfred Hospital, Australia); I Hanley (The Tweed Hospital, Australia); M Dickfos (Toowoomba Hospital, Australia); C Nawara, F Primavesi, D Öfner (Department Of Surgery, Paracelsus Medical University Salzburg, Austria); H Hakim, M Hussain, T Kumar (Dhaka Medical College Hospital, Bangladesh); K Mahmud, AR Mitul (Dhaka Shishu, Bangladesh); A Oosterkamp (Lamb Hospital, Bangladesh); PA Assouto, I Lawani, YI Souaibou (Centre National Hospitalier Et Universitaire Hubert Koutoukou Maga, Benin); VDP Castillo, G Moreira, MM Munhoz (Conjunto Hospitalar De Sorocaba, Brazil); MC Careta, SAK Ferreira, LCB De Castro Segundo (Hospital Da Santa Casa De Misericórdia De Vitória, Brazil); ADL Cury, SB Kim, AV De Sousa (Hospital De Caridade Sãƒo De Paula, Brazil); GP Fraga, DVD Santos, RL Simoes (Hospital De Clinicas, University Of Campinas, Brazil); GPS Miguel, BP Silvestre, AVC De Freitas (Hospital Estadual Doutor Jayme Dos Santos Neves, Brazil); CO Felipe, LAV Laufer, JGP Vianna (Hospital Estadual Doutor Jayme Santos Neves, Brazil); F Altoe, TF Giuriato, JS Luiz, PAB Morais, ML Pimenta, LAD Silva (Hospital Estadual Dr Jayme Santos Neves, Brazil); R Araujo, A Leal, M Leal, J Menegussi, LS Tatagiba, CVB De Lima (Hospital Infantil Nosa Senhora Da Gloria, Brazil); CL Chong, AK Tun (Pmmpmhamb Hospital, Brunei); KP Aung, CL Chong, LS Yeo (Ripas Hospital, Brunei); CL Chong, GH Devadasar, MRM Qadir (Ssb Hospital, Brunei); S Stock (World Mate Emergency Hospital, Cambodia); J Brown, J Kabba, TE Ngwa, S Nigo (Mbingo Baptist Hospital, Cameroon); DL Deckelbaum, A Horobjowsky, T Razek (Centre for Global Surgery, McGill University Health Centre, Canada); K Bailey, B Cameron, M Livingston (McMaster Children's Hospital, Canada); A Agarwal, G Azzie, M Firdouse, S King, S Kushwaha, A Zani (The Hospital For Sick Children, Canada); N D'aguzan, E Grasset, B Marinkovic (Hospital Del Salvador, Chile); E Grasset, J Jimenez, R Macchiavello (Hospital Luis Tisne, Chile); W Guo, J Oh, Z Zhang, F Zheng (Beijing Friendship Hospital, China); M Mendez, I Montes, S Sierra (Clinica Ces, Colombia); MCM Arango, I Mendoza, MI Villegas (Clinica Las Vegas, Colombia); FAN Aristizã¡bal, JAM Botero, VMQ Riaza (Hospital Pablo Tobon Uribe, Colombia); MCM Arango, C Morales, J Restrepo (Hospital Universitario San Vicente Fundacion, Colombia); MCM Arango, H Cruz, A Munera (Ips Universitaria Clinica Leon Xiii, Colombia); N Pezelj, M Radic, K Zamarin (General Hospital Sibenik, Croatia); E Domini, R Karlo, J Mihanovic (Zadar General Hospital, Croatia); M Hache-Marliere, SB Lemaire, R Rivas (Cedimat - Centro De Diagnostico Medicina Avanzada, Laboratorio Y Telemedicina, Dominican Republic); MAB Fahmy, A Hassan, A Khyrallh, G Shimy (Al-Azher Universty Hospital, Egypt); I AbdelFattah, M Abdulgawad, M Abozaid, A Adel, A Al-Mallah, M Alhendy, M Baheeg, A Elgebaly, AE Elshafay, AA Fattah, M Gemeah, A Gharib, A Gharib, A Gouda, M Hanafy, A Hasan, A Kenibar, A Menshawy, A Mohammed, A Mohammed, O Osman, O Saleh, A Sayed (Al-Hussein Hospital, Egypt); M Abdelkader, M Asal, M Elfil, M Ghoneem, MEAM Gohar, A Gomaa, M Gomah, M Karkeet, A Nabawi, H Rashwan (Alexandria Main University Hospital, Egypt); O Alahmady, A Alkammash, AAA Ata, AM Attia, AA El Galeel, NA El Hamid, KS El-Dien, E Elbanby, AM Elkorashy, U Hantour, AHE Kotb, B Mansour, M Nasr, M Saeed (Bab El-Shareia Hospital, Egypt); NYE Abdel-Wahab, MAF Abozyed, A Adel, GS El Sayed, SS Elkolaly, KT Lasheen, AM Saeed, EMS Taha, JH Youssif (Banha University Hospital, Egypt); SM Ahmed, NS El-Shahat, AEH Khedr (Belbes Central Hospital, Egypt); AM Afifi, OS Ebrahim, MM Metwally (El - Mataria Educational Hospital, Egypt); M Abbas, M Abdelraheim, KN El Deen, AE Elnemr, AO Elsebaaye, I Elzayat, M Elzayat, I Elzayyat, D Hemeda, H Khaled, M Rashad, O Salah, M Salama, M Seisa, G Tawfik, M Warda (El Dawly Hospital - Mansoura, Egypt); M Elkhadrawi, K Elshaer, A Hussein (El Mahalla General Hospital, Egypt); A Abdelgelil, S Abdelghany, A Aboarab, M Aboraya, AA Al-Aarag, A El Kholy, M Elbermawy, F Elkady, A Elkholy, R Elmelegy, DME Elsawahly, S Elshanwany, R Fakher, AA Ghazy, A Haroun, E Nofal, H Safa, A Sakr, M Salma, H Samih, A Samir, S Samy (El-Menshawy Hospital, Egypt); E Ghanem (Elshohadaa Central Hospital, Egypt); G El Ashal, Y El Shoura, AM Hammad, H Khairy, A Tammam (Kasr Alainy School Of Medicine, Egypt); E Abdallah, M Abdelshafy, A Abouzahra, T Alzayat, S Antar, H Elfeki, FI Elgendy, S Elsheikh, E Gamaly, MGM Hamad, M Hosh, B Magdy, S Mehrez (Mansoura University Hospitals, Egypt); Y Abd-Elrasoul, M Abuseif, M Alrahawy, M Ammar, MS Ammar, SAE Barakat, FA El-Salam, A Elkelany, A Elkelany, YA Elrasoul, N Elsayed, H Elwakil, M Etman, A Eysa, Y Hegazy, M Morsi, M Mustafa, A Nasr, A Raslan, A Rslan, S Saad, A Sabry, A Sadek, O Seifelnasr, H Shaker, AG Toeema, H Zidan, H Zidan (Menoufia University Hospitals, Egypt); H El-Kashef, M Shaalan, A Tarek (Minia University Hospital, Egypt); A Almallah, A Elwan, A Elwan, D Emadeldin, A Fouad, MA Ghonaim, AR Nayel, EA Sayma, M Seif (New Damietta University Hospital, Egypt); OSA El Hameed, AS El-Ma'doul, A Elbatahgy, DEAA Elsorogy, A Lasheen, A Mosad, HA Mostafa, AA Omar, H Tolba (Quweisna Central Hospital In Quweisna, Egypt); YA El Salam, M Ismail, A Morsi (Ras El Tin General Hospital, Egypt); A Abouelnasr, A Afandy, MA Amer, M Amreia, NA Attallah, S Ayad, AA El Magd, AS El-Hamouly, HA El-badawy, A Elkelany, A Elkelany, S Elsobky, AT Hafez, A Marey, A Mokhtar, O Mosalum, M Mustafa, R Sakr, R Shaker, R Shaker, MF Zalabia (Shebin Elkom Teaching Hospital, Menoufia, Egypt); EA Ahmed, A Fadel, MM Mohamed (Sohag University Hospital, Egypt); I AlYoussef, A Aldalaq, A Ali, D Alkhabbaz, E Alnawam, MG Alwafai, AK Aly, A Dwydar, H El-Sheemy, S Kharsa, E Mamdouh (Souad Kafafi University Hospital, Egypt); M Elashmawy, AA Elazayem, I Elkadsh, ZM Elsayed, A Elwaey, S Ghanem, S Hussein, A Meshref, M Mousa, A Nashaat, M Saad (Suez Canal University Hospitals, Egypt); M Darweesh, M Hafez, A Mohameden (Suez General Hospital, Egypt); A Badr, A Badwy, MA El Slam (Talla Central Hospital, Egypt); A Abdelkareem, M Aboraya, K Abozeid, S Al-Nahrawi, M Allam, M Ameen, S Aql, H Dawoud, A El Gendy, S El Mesery, M Elazoul, L Eldamaty, AOA Elhendawy, M Elsehimy, M Elshobary, A Fahiem, A Hagar, A Hashish, M Hashish, AS Marey, F Nada, S Sarsik, S Shehata, M Zidan (Tanta University Hospital, Egypt); NM Badwi, N Elfouly, Y Elfouly, AS Elsherbiny, A Fawzy, A Gheith, MA Habeeb, A Hassan, M Husseini, Y Ibrahim, E Kasem, O Mohamed, MMH Mohammed, M Rashid, B Sieda, AR Soliman (Zagazig University Hospitals, Egypt); N Starr, M Worku (Dessie Referral Hospital, Ethiopia); NS Abebe, S Desta, S Wondimu (Minilik Ii Hospital, Ethiopia); FA Asele, D Dabessa, E Thomas (Myungsung Christian Medical Center, Ethiopia); NS Abebe, AB Zerihun (Tikur Anbessa Hospital, Ethiopia); A Leppäniemi, P Mentula, V Sallinen (Helsinki University Central Hospital, Finland); Q Alimi, E Gaignard, V Graffeille (Chu De Rennes, France); Q Alimi, E Gaignard, V Graffieille (Fr Rennes University, France); O Abbo, O Bouali, S Mouttalib (Hopital Des Enfants, France); Y Aigrain, N Botto, E Hervieux (Hopital Necker Enfants Malades, France); A Faure, L Fievet, N Panait (Hopitâl Nord, France); E Eyssartier, G Podevin, F Schmitt (Pediatric Surgery Department, University Hospital Of Angers, France); AP Arnaud, A Martin, V Parent (Rennes University Hospital, France); A Bonnard, C Muller, M Peycelon (Robert Debré Children University Hospital, France); F Frade, S Irtan, A Scalabre (Trousseau Hospital Sorbonnes Universités, Upmc Univ Paris, France); F Abantanga, K Boakye-Yiadom, M Bukari (Komfo Anokye Teaching Hospital, Ghana); F Owusu (Offinso District Hospital, Ghana); J Awuku-Asabre, LD Bray, S Tabiri (University For Development Studies, School Of Medicine And Health Sciences, General Surgery Department,Tamale Teaching Hospital, Ghana); A Bamicha, D Lytras, K Psarianos (Achillopoyleio General Hospital Of Volos, Greece); E Kefalidi (Attikon General Hospital, Greece); G Gemenetzis (Attikon University Hospital, Greece); C Agalianos, C Dervenis, N Gouvas (Konstantopouleio General Hospital Of Athens, Greece); D Karousos, M Kontos, G Kouraklis (Laiko University Hospital, Greece); S Germanos, C Marinos (Larissa General Hospital, Greece); C Anthoulakis, N Mitroudis, N Nikoloudis (Serres General Hospital, Greece); S Estupinian, W Forno, G Recinos (Hospital De Accidentes Ceibal, Guatemala); JRA Azmitia (Hospital General De Enfermedades, Cirugia De Emergencia, Instituto Guatemalteco De Seguridad Social, Guatemala); CCR Cabrera (Hospital General De Enfermedades, Servicio De Cirugia Abdominal, Instituto Guatemalteco De Seguridad Social, Guatemala); M Aguilera, R Guevara, N Mendez, CAA Mendizabal, P Ramazzini, MC Urquizu (Hospital General San Juan De Dios, Guatemala); E Barrios, E Barrios, R Soley, F Tale (Hospital Juan Jose Arevalo Bermejo, Guatemala); SMC Mérida, DEM Rodríguez, CIP Velásquez (Hospital Regional De Retalhuleu, Guatemala); M Lopez, F Regalado, M Siguantay (Hospital Roosevelt, Guatemala); FY Lam, MF Leung, KKK Li, WS Li, T Mak, S Ng, CCL Szeto, KJ Szeto (Prince of Wales Hospital, Hong Kong); N Gyanchandani, A Kirishnan, SS Prasad (KMC Hospital, India); S Bhat, SV Kinnera, A Sreedharan (Kasturba Medical College, India); BS Kumar, M Rangarajan (Kovai Medical Centre & Hospital, India); S Kumar, Y Reddy, C Venugopal (Pes Institute Of Medical Sciences & Research, India); A Mittal (Safdarjung Hospital,New Delhi, India); HN Lakshmi, P Malik, S Nadkarni (Sawai Man Singh Medical College & Hospitals, Jaipur, Rajasthan, India); P Jain, N Limaye, S Pai (Sdm College Of Medical Sciences And Hospital, India); M Khajanchi, R Satoskar, S Satoskar (Seth Gordhandas Sunderdas Medical College And King Edward Memorial Hospital, India); AB Mahamood (Travancore Medical College Hospital, India); DA Soeselo, EPR Sutanto, C Tedjaatmadja (Atmajaya Hospital, Indonesia); R Amandito, M Mayasari, FN Rahmawati (Dr Cipto Mangunkusumo General Hospital, Jakarta, Indonesia); IAA Al-Azraqi, HII Al-Hameedi, RKMJ Al-Hasani, HI Ibraheem (Alsader Medical City, Iraq); R Kamil, L Sabeeh, M Shawki (Baghdad Medical City, Iraq); MM Telfah (Department Of Surgery, College Of Medicine, University Of Mosul, Al-Jumhoori Teaching Hospital, Iraq); S Gosling, M Mccarthy, A Rasendran (Cork University Hospital & University College Cork, Ireland); M Dablouk, MO Dablouk, RW Gilbert, M Hanrahan, R Kerley, P Kielty, E Marks, L Mauro, C Normile, A Rasendran, J Sheehan, J Song (Cork University Hospital, Ireland); D Mirghani, SA Naqvi, CS Wong (Limerick University Hospital, Ireland); R Cahill, S Chung, R D'cruz (Mater Misericordiae University Hospital, Ireland); DD Cadogan, C Clifford, A Driscoll, C Fahy, R Gilbert, SG Gosling, M Hanrahan, M Mccarthy, C Normile, A Powell, A Rasendran, J Song (Mercy University Hospital, Ireland); R Bowe, C Lee, S Paul (Midlands Regional Hospital Mullingar, Ireland); M Hanrahan, W Hutch (University College Cork, Ireland); K Mealy, H Mohan, M O'neill (Wexford General Hospital, Ireland); A Bondurri, P Danelli, A Maffioli (Azienda Ospedaliera Luigi Sacco - Polo Universitario, Italy); M Pasini, G Pata, S Roncali (Azienda Ospedaliera Spedali Civili Di Brescia - Chirurgia Generale, Italy); M Carlucci, R Faccincani, P Silvani (Irccs Ospedale San Raffaele, Italy); K Khattab, G Tugnoli, S Di Saverio (Maggiore Hospital, Italy); LM Cloro, MA Paludi, D Pata (Nicola Giannettasio Hospital, Italy); A Allegri, L Ansaloni, F Coccolini (Papa Giovanni Xxiii Hospital, Italy); L Bortolasi, A Hasheminia, E Veronese (San Bonifacio Hospital, Italy); A Benevento, F Pata, G Tessera (Sant'Antonio Abate Hospital, Gallarate, Italy); MD Canto, S Cucumazzo, G Nastri (Santa Croce Hospital, Italy); PP Grandinetti, GL Lamanna, A Maniscalco (Santi Benvenuto E Rocco Hospital Asur, Italy); E Rausa, G Sgroi, L Turati (Treviglio Hospital, Italy); A Allegri, L Ansaloni, F Coccolini (Unit Of General Surgery I, Papa Giovanni Xxiii Hospital, Italy); D Merlini, M Monteleone, R Villa (Unita' Di Chirurgia D'urgenza Azienda Ospedaliera Salvini, Italy); A Cacurri, R Cirocchi, V Grassi (University Of Perugia, Italy); L Bonavina, C Ceriani, Y Macchitella (University of Milan, IRCCS Policlinico San Donato, Italy); A Diab, F Elzowawi, H Waleed (Misurata Central Hospital, Libya); M Jokubauskas, K Varkalys, D Venskutonis (Kaunas Clinical Hospital, Lithuania); V Ambrozeviciute, R Pranevicius (Klaipedas Seaman Hospital, Lithuania); S Juciute, A Skardžiukaitė (Lietuvos Sveikatos Mokslų Universitetas, Lithuania); A Austraite, S Bradulskis, Z Dambrauskas, R Riauka, L Urbanavicius, D Venskutonis, J Zilinskas (Lithuanian University Of Health Sciences, Lithuania); P Karumnas, Z Urniezius, R Zilinskiene (Republic Hospital Of Kaunas, Lithuania); A Rudzenskaite (Republic Hospital Of Panevezys, Lithuania); N Kaselis, M Montrimaite, A Usaityte (Republic Klaipeda Hospital, Lithuania); K Jokubonis, A Strazdas (Stasys Kudirka Regional Hospital Of Alytus, Lithuania); V Jotautas, A Kolosov, I Rakita (Vilnius University Hospital Santariskiu Clinics, Lithuania); V Beisa, D Kazanavicius, S Mikalauskas, T Poskus, R Rackauskas, K Strupas (Vilnius University Hospital Santariskiu Klinikos, Lithuania); V Beisa, E Laugzemys, K Maceviciute, T Poskus, K Strupas (Vilnius University, Center Of Abdominal Surgery, Lithuania); E Preckailaite, R Rakauskas (Vsi Jonavos Ligonine, Lithuania); R Coomber, K Johnson, J Nowers (Queen Elizabeth Hospital, Malawi); A Das, D Periasammy, A Salleh (Hospital Kajang, Malaysia); NAN Abdullah, MN Kumar, RGE Tze (Sarawak General Hospital, Malaysia); NR Kosai, R Rajan, M Taher (Universiti Kebangsaan Malaysia Medical Centre, Malaysia); HY Chong, CC Goh, AC Roslani (University Malaya Medical Centre, Malaysia); M Agius, M Bezzina, E Borg, R Bugeja, J Psaila, A Spina, M Vella-Baldacchino (Mater Dei Hospital, Malta, Malta); J Colombani, H Francois-Coridon, C Tolg (Department Of Pediatric Surgery, Mother And Children's Hospital, University Hospital Of Martinique, Martinique); C Diaz-Zorrilla, SC Gonzalez, A Ramos-De la Medina (Hospital Español de Veracruz, Mexico); M Jacobe, D Mapasse, E Snyder (Hospital Central Maputo, Mozambique); M Osman, R Oumer (Whangarei Hospital, Northland District Health Board, New Zealand); L Anyanwu, A Mohammad, A Sheshe (Aminu Kano Teaching Hospital, Nigeria); A Adesina, O Faturoti, O Taiwo (Babcock University Teaching Hospital, Nigeria); MH Ibrahim, AA Nasir, SI Suleiman (Federal Medical Centre, Birnin Kebbi, Nigeria); A Adebanjo, A Adeniyi, O Adesanya (Federal Medical Centre, Nigeria); K Atobatele, A Ogunyemi, M Oludara, O Oshodi, R Osuoji, O Williams (Lagos State University Teaching Hospital, Nigeria); A Ademuyiwa, F Alakaloko, C Bode, O Elebute, AO Lawal, A Osinowo (Lagos University Teaching Hospital, Nigeria); A Adesuyi (National Hospital Abuja, Nigeria); A Adekoya, C Nwokoro, A Tade (Olabisi Onabanjo University Teaching Hospital, Nigeria); AE Ajao, OO Ayandipo, TA Lawal (University College Hospital, Nigeria); SS Ali, B Odeyemi, S Olori (University of Abuja Teaching Hospital, Nigeria); J Adeniran, A Adeyeye, A Popoola (University of Ilorin Teaching Hospital, Nigeria); WJ Lossius (Department Of Gastrointestinal Surgery, St. Olavs Hospital, Trondheim University Hospital, Norway); I Havemann (Soerlandet Hospital Kristiansand, Norway); JK Narvestad, K Soreide, K Thorsen (Stavanger University Hospital, Norway); L Nymo, TB Wold (University Hospital Of North Norway, Troms, Norway); M Dar, M Elsiddig (Sohar Hospital, Oman); KF Bhopal, MM Furqan, Z Iftikhar (Bahawal Victoria Hospital, Pakistan); M Jawaid, A Khalique, B Nighat (Dow University Hospital, Pakistan); A Rashid, A Zil-E-Ali (Fatima Memorial Hospital, Pakistan); HA Dharamshi, A Faraz, T Naqvi (Karachi Medical And Dental College, Abbasi Shaheed Hospital, Pakistan); AW Anwar, W Anwer, G Shamsi, GS Shamsi, T Yaseen, TM Yaseen (The Indus Hospital, Pakistan); O Aguilera, IIZ Alvarez, HP Decoud, JM Delgado, HAS Lohse, GMM Vega (Hospital De Clínicas, Paraguay); WLM Aguilar, ACM Bautista, JAC Chiong (Carlos Alberto Seguin Escobedo National Hospital. Essalud, Peru); JMV Celis, DAR Pozo (Hospital De Emergencias Pediatricas, Peru); J Hamasaki, J Herrera-Matta, E Temoche (Hospital De Policia, Peru); LMA Barreda, RRB Ojeda, CPG Torres (Hospital Goyeneche, Peru); O Garaycochea (Hospital Ii -1 Minsa Moyobamba, Peru); F Fujii, MC Mollo, MS De Fã Tima Linares Delgado (Hospital Maria Auxiliadora, Peru); WLM Aguilar, ACM Bautista, JAC Chiong (Hospital Nacional Carlos Alberto Seguin, Peru); MR Castro, RA Jaramillo, GB Luque, AER Moran, ALC Vergara, SBS Yip (Hospital Nacional Cayetano Heredia, Peru); CAA Basto, SYA Durand, NMU Rojas (Hospital Nacional Edgardo Rebagliati Martins-Essalud, Peru); R Camacho, E Huaman, S Zegarra (Hospital Necional Guillermo Almenara, Peru); A Arenas, C Hinojosa, RC Huaraya, S Limache, CE Lopez, C López, M Machaca, W Pino, CMH Puma, LM Rodriguez, GM Sila, GM Sila, MZP De Leon, MZP De Leon (Hospital Regional Honorio Delgado Espinoza, Peru); J Costa-Maia, R Melo, N Muralha (Servico De Cirurgia Geral - Centro Hospitalar Sao Joao - Porto, Portugal); F Sauvat (Chu Reunion, Reunion); I Dan, P Eduard, M Hogea (Emergency Clinical Hospital Brasov, Romania); M Beuran, R Bratu, I Diaconescu, F Iordache, B Martian, M Vartic (Emergency Clinical Hospital Bucharest, Romania); AS Mironescu, LI Muntean, LC Vida (Spitalul Clinic De Copii Brasov, Romania); VJP Nsengimana (Chuk, Rwanda); A Niragire, E Niyirera, J De La Croix Allen Ingabire (University Teaching Hospital Of Kigali, Rwanda); E Jovine, G Landolfo, N Zanini (San Marino State Hospital, San Marino); SA Alnuqaydan, IN Alomar, AM Altwigry (Buraydah Central Hospital, Saudi Arabia); N Akeel, M Aljiffry, M Alsaggaf, A Altaf, M Bakhaidar, A Habeebullah, A Khoja, AA Maghrabi, A Nawawi (Department of Surgery, Faculty of Medicine, King Abdulaziz University, Jeddah, Saudi Arabia); M AlRowais, A Althwainy (Department of Surgery, King Saud University, Saudi Arabia); N Osman, M Othman (Imam Abdulrahman Al Faisal Hospital, Saudi Arabia); E Alqahtani (King Abdulaziz Hospital Al Ahsa National Guard, Saudi Arabia); E Aljohani, R Alyami, M Alzahrani (King Abdulaziz Medical City, Riyadh, Saudi Arabia); I Alhabli, E Aljohani, S Almuallem, R Alyami, M Alzahrani, Z Mikwar (King Abdulaziz Medical City, Saudi Arabia); A Almoflihi, N Ghandora, A Huwait (King Fahad General Hospital, Saudi Arabia); M Al-Mousa, A Al-shammari (King Fahad Hospital, Saudi Arabia); W Adham, S Al Awwad, B Albeladi, MA Alfarsi, M Alghamdi, A Mahdi (King Fahd Hospital, Saudi Arabia); A Altamimi, M Hassanain, T Nouh (King Khaled University Hospital, King Saud University, Saudi Arabia); S Aldhafeeri, O Algohary, N Sadig (King Khalid General Hospital, Saudi Arabia); M Aledrisy, A Alrifaie, A Gudal (King Khalid National Guard Hospital, Saudi Arabia); U Alamoudi, M Alrajraji, A Shabkah (National Guard Hospital, Saudi Arabia); B Alghamdi, S Aljohani, A Daqeeq (Rcymc, Saudi Arabia); JJ Al-Faifi (Security Forces Hospital, Saudi Arabia); V Jennings, R Moore, N Ngayu (Chris Hani Baragwanath Academic Hospital, South Africa); V Kong (Edendale Hospital, South Africa); K Connor, H Kretzmann, D Nel (Frere Hospital, South Africa); E Panieri, C Sampson, R Spence (Groote Schuur, South Africa); S Rayne, N Sishuba (Helen Joseph Hospital, Department Of Surgery, University Of The Witwatersrand, South Africa); J Carreira, AM Mphatsoe, M Tun (Leratong Hospital, South Africa); E Teasdale, M Wagener (Ngwelezana Hospital, South Africa); S Botes, D Du Plessis (Rob Ferreira Hospital, South Africa); F Fernandez-Bueno (Hospital Central De La Defensa Gomez Ulla, Spain); J Aguilar-Jimenez, JA Garcia-Marin (Hospital Morales Meseguer. Sms, Spain); LJG Florez, LS García, RDA Pacheco (Hospital San Agustín, Spain); L Barneo, C Lopez-Arevalo, G Minguez, J Pagnozzi, JHJ Quezada, JL Rodicio, R Rodríguez-Uría, JPG Stuva, P Ugalde (Hospital Universitario Central De Asturias, Spain); N Herrera, I Ortega-Vazquez, L Rodriguez (Severo Ochoa University Hospital, Spain); PP Arachchi, LAJJ Arachchige, WSMKJ Senanayake (Department Of General Surgery, Teaching Hospital Kandy, Sri Lanka); DI Samaraweera, S Sivaganesh, V Thanusan (National Hospital Of Sri Lanka, Sri Lanka); RMH Balila, MAEH Mohamed, AEK Musa (Ibrahim Malik Teaching Hospital, Sudan); H Ali, HZ Elabdin, A Hassan (Jarash International Specialized Hospital, Sudan); H Ahmed, SAI Idris, S Mahdi (Khartoum Teaching Hospital, Sudan); M Elsayed, M Elsayed, M Mahmoud (Omdurman Teaching Hospital, Sudan); M Boijsen, P Lundgren (Capio St Goran Hospital, Sweden); U Gustafsson, A Kiasat (Danderyds Hospital, Sweden); E Jurdell, A Thorell, F Wogensen, F Wogensen (Department Of Surgery, Ersta Hospital, Stockholm, Sweden); L Andersson, U Gunnarsson, M Sund (Department Of Surgical And Perioperative Sciences, Umea University and Umea University Hospital, Sweden); H Thorarinsdottir, M Utter (Helsingborgs Lasarett, Sweden); SM Sundstrom (Hudiksvall Sjukhus, Sweden); A Kjellin, C Wredberg (Karolinska Universitetssjukhuset, Sweden); B Frisk, J Nyberg (Skaraborg Hospital Skovde, Sweden); S Ahlqvist, I Björklund, Y Cengiz (Sundsvall Hospital, Sweden); H Royson, P Weber (Vaexjoe Central Hospital, Sweden); H Royson, P Weber (Vaxjo Central Hospital, Sweden); E Borin, H Pahlsson (Visby Hospital, Department Of Surgery, Sweden); M Hjertberg (Vrinnevi Hospital, Sweden); V Despotidis, D Schivo, R Schmid (Bürgerspital Solothurn, Switzerland); F Deichsel, A Gerosa, A Nocito (Kantonsspital Baden, Switzerland); L Eisner, B Mijuskovic, DA Raptis, M Zuber (Kantonsspital Olten, Switzerland); S Breitenstein, E Schadde, RF Staerkle (Kantonsspital Winterthur, Switzerland); S Kruspi, KB Reinisch, C Schoewe (Kreisspital Fuer Das Freiamt Muri Ag, Switzerland); A Novak, AF Palma, G Teufelberger (Kreisspital Muri, Department Of Surgery, Switzerland); M Kimaro, R King (Mbalizi Christian Designated Hospital, Tanzania); AZA Balkan, M Gumar, MA Yavuz (Harran University Research And Treatment Hospital, Turkey); U Karabacak, G Lap, BB Ozkan (Ondokuz Mayis University, Medical Faculty, Turkey); M Karakahya, BB Ozkan (Ordu University Training And Research Hospital, Turkey); R Adams, KY Chang, KD Clement, R Gratton, L Henderson, R Mcintosh, D Mcnish, W Milligan, R Morton (Aberdeen Royal Infirmary, United Kingdom); H Anderson-Knight, R Lawther, B Skelly (Altnagelvin Area Hospital, United Kingdom); J Onimowo, V Shatkar, S Tharmalingam (Barking, Havering And Redbridge University Hospitals Trust, United Kingdom); T Fautz, E Woin, O Ziff (Barnet General Hospital, United Kingdom); S Arman, S Arman, S Dindyal, V Gadhvi, S Talukder, S Talukder (Basildon And Thurrock University Foundation Trust, United Kingdom); LS Chew, J Heath (Blackpool Victoria Teaching Hospitals, United Kingdom); N Blencowe, K Gash, S Hallam (Bristol Royal Infirmary, United Kingdom); GS Mannu, AC Snaith, D Zachariades (Buckinghamshire NHS Trust, United Kingdom); TS Hettiarachchi, A Nesaratnam, J Wheeler (Cambridge University Hospitals NHS Foundation Trust, United Kingdom); JM Clements, A Khan, D McCullagh (Causeway Hospital, United Kingdom); A Ahmed, JLY Allen, J Almy, A Ashton, M Deputy, T Khan, F Koumpa, DC Marshall, CJ Mcintyre, C Neophytou, J Roth, WC Soon, J Vincent (Charing Cross Hospital, Imperial College Healthcare NHS Trust, United Kingdom); N Behar, H Jordan, M Sykes (Chelsea And Westminster Hospital, United Kingdom); Y Rajjoub, T Sherman (Cheltenham General Hospital, United Kingdom); R Ardley, A Watts, T White (Chesterfield Royal Hospital NHS Foundation Trust, United Kingdom); T Arulampalam, D Brown, A Shah (Colchester Hospital University NHS Foundation Trust, United Kingdom); E Blower, K Gasteratos, P Sutton, D Vimalachandran (Countess Of Chester Hospital, United Kingdom); G Irwin, C Magee, A Mcguigan (Craigavon Area Hospital, United Kingdom); S Mcaleer, C Morgan (Daisy Hill Hospital, United Kingdom); S Braungart (Department Of Paediatric Surgery, Leeds General Infirmary, United Kingdom); P Labib, K Lafferty, C Mangan, C Mangan, L Reza, L Reza, A Tanase, A Tanase (Derriford Hospital, United Kingdom); C Gouldthorpe, M Turner, H Woodward (Diana Princess Of Wales Hospital, United Kingdom); TAM Malik, VK Proctor, JRL Wild (Doncaster Royal Infirmary NHS Foundation Trust, United Kingdom); J Davies, K Hewage (Dorset County Hospital, United Kingdom); A Dubois, A Grant, R Mcintyre, S Sarwary, A Zardab (Dr Grays Hospital, United Kingdom); BFHK Chong, W Ho, YP Mogan (Dumfries And Galloway Royal Infirmary, United Kingdom); E Farinella, G Humm, S Tewari (East And North Hertfordshire NHS Trust Lister Hospital, United Kingdom); NJ Hall, CP Major, NJ Wright (Evelina Children's Hospital, United Kingdom); J Amin, J Amin, M Attard, M Baldacchino, H Burns, JF Camilleri-Brennan, J Camilleri-Brennan, M Farhad, A Jabbar, E Macdonald, J Richards, AGN Robertson, J Skehan, J Swann, T Xerri, T Xerri, P De Bono, P De Bono (Forth Valley Royal Hospital, United Kingdom); M Gimzewska, TF Hall, G Mclachlan (Frimley Park Hospital, United Kingdom); J Giles, J Shah (George Elliot Hospital, United Kingdom); S Chiu, SMY Chiu, S Highcock, B Weber (Gilbert Bain Hospital, United Kingdom); W Beasley, S Dias, M Hassan, G Maharaj, R Mcdonald, A Vlachogiorgos (Glangwili General Hospital, United Kingdom); A Baird, A Macdonald, P Witherspoon (Glasgow Southern General Hospital, United Kingdom); N Green, P Sarmah, H Youssef (Good Hope Hospital, United Kingdom); K Cross, CM Rees, B Van Duren (Great Ormond Street Hospital For Children NHS Foundation Trust, United Kingdom); E Upchurch (Great Western Hospital, United Kingdom); H Abudeeb, A Hammad, K Khan (Hairmyres Hospital, NHS Lanarkshire, United Kingdom); D Bowley, S Karandikar, A Karim (Heart Of England Foundation Trust, United Kingdom); O Al-Obaedi, A Bhangu, W Chachulski, K Das, G Dawnay, M Ghetia, M Ghetia, A Mistry, L Richardson, S Roy, B Thompson (Hereford County Hospital, United Kingdom); DM Cocker, A Prabhudesai, JJ Tan (Hillingdon Hospital, United Kingdom); S Ayyar, R Tyler, F Di Franco (Hinchingbrooke Hospital, United Kingdom); S Gokani, S Vivekanantham (Imperial College London, United Kingdom); M Gillespie, K Gudlaugsdottir (Inverclyde Royal Hospital, United Kingdom); C Currow, MY Kim, T Pezas (Ipswich Hospital NHS Trust, United Kingdom); A Ali, K Atkinson, A Birring, S Das, J Edwards, M Jha (James Cook University Hospital, United Kingdom); T Fozard, J Luck, M Puttick (John Radcliffe Hospital, United Kingdom); H Ebdewi, S El-Rabaa, G Gravante, AA Ibrahem, Y Salama, R Shah (Kettering General Hospital, United Kingdom); R Allott, A Bhargava, H Nnajiuba (King George Hospital, United Kingdom); Z Chan, Z Hassan (Kings College Hospital, United Kingdom); A Aber, A Boddy, R Dean, D Hemingway, M Makinde, V Patel (Leicester Royal Infirmary, United Kingdom); J Parakh (Leighton Hospital - Mid Cheshire NHS Foundation Trust, United Kingdom); S Parthiban (Lister Hospital, United Kingdom); S Hosein, HK Ubhi (Luton And Dunstable Hospital, United Kingdom); K Malik, S Ward (Macclesfield District General Hospital, United Kingdom); M Alkhouri, J Barry, C Houlden, L Jennings, MK Kang, T Newton (Morriston Hospital, United Kingdom); S Bhattacharya, A Farquharson, I Raza (NHS Ayrshire, United Kingdom); K Chang, L Henderson, W Milligan (NHS Grampian, United Kingdom); R Blundell, E Chan, I Ibrahim, PJ Lim, YN Neo, AS North, FS Peck, A Williamson, MSJ Wilson (Ninewells Hospital, NHS, United Kingdom); D Fouad, A Minocha (Norfolk And Norwich University Hospital, United Kingdom); A Chambers, E Court, K Mccarthy (North Bristol NHS Trust, United Kingdom); C Beaton, JC Tham, J Yee (North Devon District Hospital, United Kingdom); S Bokhari, M Griffiths, L Howells, J Lockey, U Walsh, L Yallop (Northwick Park Hospital, United Kingdom); P Jackson, O Nasher, S Singh (Nottingham Children's Hospital At Queens Medical Centre Campus, United Kingdom); T Fozard, J Luck, M Puttick (Oxford University Hospitals, United Kingdom); WC Ho, G Pabla, AM Shariffuddin, MS Wilson (Perth Royal Infirmary, United Kingdom); J Doughty, S Ramzi, S Zeidan (Plymouth Hospitals NHS Trust, United Kingdom); R Davenport, J Lewis, S Sinha (Princess Alexandra Hospital, United Kingdom); L Duffy, E Mcaleer, E Williams (Princess Of Wales Hospital, United Kingdom); M Boal, T Brogden, E Griffiths, N Harrison, O Javed, D Nepogodiev, H Tafazal (Queen Elizabeth Hospital Birmingham, United Kingdom); DJ Clark, TE Glover, RD Obute (Queen Elizabeth Hospital King's Lynn, United Kingdom); O Javed, R Som (Queen Elizabeth Hospital Woolwich, United Kingdom); M Akhtar, M Boshnaq, P Capleton, S Doughan, I Mohamed, M Rabie (Queen Elizabeth The Queen Mother Hospital, United Kingdom); E Brown, E Dempster, L Dickson, A Garland, M Kennedy, N Maple, E Monaghan, D Samuel, B Wolf (Raigmore Hospital, United Kingdom); D Anderson, R Anderson, A Mcphee (Royal Alexandria Hospital, United Kingdom); S Hassan, D Smith, P Sutton (Royal Bolton Hospital, United Kingdom); C Boereboom, J Lund, J Murphy, G Tierney, S Tou (Royal Derby Hospital, United Kingdom); I Daniels, K Findlay-Cooper, T Stasinou (Royal Devon & Exeter NHS Foundation Trust, United Kingdom); NJ Smart, AM Warwick, EF Zimmermann (Royal Devon And Exeter National Health Service Foundation Trust, United Kingdom); R D'Souza, S Mitrasinovic, S Omara, S Ray, M Varcada (Royal Free Hospital, United Kingdom); A Hanks, L Parkinson, M Spurr (Royal Glamorgan Hospital, United Kingdom); E Abington, J Ma, M Ramcharn, G Williams (Royal Gwent Hospital, United Kingdom); ED Kennedy, J Winstanley, ENW Yeung (Royal Hospital For Sick Children, United Kingdom); C Fairfield, C Fairfield, SJ Fergusson, C Jones, S Koh, I Liew, SJ Lim, H Nair, S O'neill, J Oh, A Wilson (Royal Infirmary Of Edinburgh, United Kingdom); D Anandkumar, SF Ashraf, S Basson, C Chandrakumar, AJ Fowler, TF Jones, A Kirupagaran, SM Lakhani, AL Mclean, P Patel, HD Torrance (Royal London Hospital, United Kingdom); J Batt, N Benons, C Bowman, M Stoddart (Royal United Hospital Bath, United Kingdom); R Harrison, C Mason, J Quayle (Salford Royal NHS Foundation Trust, United Kingdom); T Barker, E Harper, V Summerour (Sandwell And West Birmingham Hospitals NHS Trust, United Kingdom); M Hampton, C Smith (Sheffield Children's Hospital, United Kingdom); TM Drake, EG Heywood, T O'Connor, SK Pitt, AE Ward (Sheffield Teaching Hospitals NHS Foundation Trust, United Kingdom); A Chowdhury, S Hossaini, NF Watson (Sherwood Forest Hospitals NHS Foundation Trust, United Kingdom); A Chun, A Farah, D Mckechnie (Southend University Hospital, United Kingdom); H Koh, G Lim, G Sunderland (Southern General Hospital, United Kingdom); DRL Browning, PC Munipalle, H Rooney (Southmead Hospital, North Bristol NHS Trust, United Kingdom); A Chambers, L Gould (Southmead Hospital, United Kingdom); E Decker, S Giuliani, K Nemeth, B Pereira, A Shalaby (St George's Healthcare NHS Trust And University, United Kingdom); CY Chen, S Chhabra, S Chidambaram, K Kulasabanathan, A Szczap (St Mary's Hospital, United Kingdom); M Benger, J Choi, M Khalili, K Patel, S Sheth, P Singh (St Thomas' Hospital, United Kingdom); EYA Palkhi, S Shaikh, CY Tan (St. James's University Hospital, United Kingdom); J Barnacle, P Harbord, E Kostov (St. Mary's Hospital, United Kingdom); A Macfarlane, L Marples, R Thurairaja (St. Thomas Hospital, United Kingdom); K Baillie, S Hafiz, MM Palliyil, J Porter, C Raslan, M Saeed, N Soltani, M Zikry (Stockport NHS Foundation Trust, United Kingdom); T Boyce, E Jones, H Whewell (The Royal Gwent Hospital, United Kingdom); N Robertson, F Th'ng (The Royal Infirmary Of Edinburgh, United Kingdom); S Galloway, A Mirza, H Saeed (The University Hospital Of South Manchester, United Kingdom); M Afzal, G Elena, M Zakir (United Lincolnshire Hospitals - Pilgrim Hospital, United Kingdom); T Clark, C Hand, P Holton, A Livesey, P Sodde, A Sriram (University Hospital Coventry And Warwickshire, United Kingdom); IS Bharj, FM Iqbal, Y Sinha (University Hospital Of North Midlands, United Kingdom); C Jenvey, A Rotundo, R Slade (University Hospital Of North Staffordshire NHS Trust, United Kingdom); AAN Abdullah, D Donoghue, L Giacci, D Golding, S Haines, P Harrison, D Loughran, MA Sherif, A Tang, TW Tilston (University Hospital Of Wales, United Kingdom); D Kotecha (University Hospitals Leicester - Leicester Royal Infirmary, United Kingdom); P Acharya, A Chapman, M Elshaer, A Riaz, J Shalhoub, T Urbonas (Watford General Hospital, United Kingdom); C Grossart, D McMorran (Western General Hospital, United Kingdom); W Hawkins, S Loizides, M Mlotshwa (Western Sussex Hospitals NHS Trust, United Kingdom); CW Ho, K Krishna, M Orchard (Weston General Hospital, United Kingdom); EE Howie, S Khan, J Shukla, F Taylor, P Thomson (Whipps Cross University Hospital, United Kingdom); O Komolafe, L Macdonald, N Mcintyre (Wishaw General Hospital, United Kingdom); J Cragg, J Parker, D Stewart (Wrexham Maelor Hospital, United Kingdom); T Farooq, L Lintin, J Tracy (Yeovil District Hospital, United Kingdom); H Kaafarani, L Luque, G Molina (Massachusetts General Hospital, United States); R Beyene, J Sava, M Scott (Medstar Washington Hospital Center, United States); R Kennedy, M Swaroop (Northwestern Memorial Hospital, United States); IA Azodo, T Chun, D Heffernan, A Stephen (The Rhode Island Hospital, United States); V Punja, M Sion, MS Weinstein (Thomas Jefferson University Hospital, United States); N Bugaev, M Goodstein, S Razmdjou (Tufts Medical Center, United States); M Hemmila, L Napolitano, K To (University Of Michigan, United States); E Etchill, M Kesinger, JC Puyana (University of Pittsburgh Medical Center - Presbyterian Hospital, United States); E Hoogakker, E Jenner, O Todd (St Francis Hospital, Zambia)

**GlobalSurg-2 Local collaborators**: G Galiqi, B Grizhja, S Ymeri (Spitali Rajonal Shkoder, Albania); R Balmaceda, JM Carmona, CG Fermani, MM Modolo, S Villalobos (Hospital Luis C. Lagomaggiore, Mendoza, Argentina); D Antezana, S Aviles, AEM Beleño, C Costa, R Klappenbach, B Sanchez (Hospital Zonal General De Agudos Simplemente Evita, Argentina); D Cox, P Deutschmann, D Hamill, S Sandler (Alice Springs Hospital, Australia); M Ashtari, H Franco (Gold Coast University Hospital, Australia); S D'Amours, D Iyer, N Niranjan (Liverpool Hospital, Australia); D Ljuhar, R Nataraja, C Sharpin (Monash Medical Center, Australia); D Gray, M Haines (Port Macquarie Base Hospital, Australia); S Al Amin, S Alamin, R Karim, S Roy, SA Tori (Armed Forces Medical College, Bangladesh); A Faruq, M Haque, F Iftekhar (Birdem Bangladesh Institute Of Research And Rehabilitation In Diabetes Endocrine And Metabolic Disorder General Hospital, Bangladesh); TH Kanta, J Razzaque, U Salma (Dhaka Medical College And Hospital, Bangladesh); S Karim, AR Mitul (Dhaka Shishu, Bangladesh); NF Aman, MM Estee (Holy Family Red Crescent Medical College & Hospital, Bangladesh); R Jonnalagadda, M O'Shea, G Padmore (Queen Elizabeth Hospital, Barbados); D Khokha, V Khokha (City Hospital, Belarus); A Filatau, A Litvin, D Paulouski, T Shachykava, M Shubianok (Gomel Regional Clinical Hospital, Belarus); F Djivoh, F Dossou, DG Gbessi, L Ismaïl, B Noukpozounkou, DM Seto, YI Souaibou (Centre National Hospitalier Et Universitaire Hubert Koutoukou Maga, Benin); F Hodonou, KR Keke (Clinique Vignon, Benin); EYS Ahounou, T Alihonou (Hopital El Fateh, Benin); G Ahlonsou, M Dénakpo (Hospital Saint Luc, Benin); AG Bedada (Princess Marina Hospital, Botswana); V Barendegere, S Kwizera, C Nsengiyumva (Hopital Militaire De Kamenge, Burundi); P Choi, S Stock (World Mate Emergency Hospital, Cambodia); A Agarwal, G Azzie, M Firdouse, L Jamal, S Kushwaha, A Zani (The Hospital For Sick Children, Canada); T Chen, C Yip (Fudan University Affiliated Huashan Hospital, China); I Montes, S Sierra, F Zapata (Clinica CES, Colombia); MCM Arango, MIV Lanau, IM Restrepo (Clinica Las Vegas, Colombia); MCM Arango, RSR Giraldo, S Sierra (Hospital Universitario San Vicente Fundación, Colombia); E Domini, R Karlo, J Mihanovic (Zadar General Hospital, Croatia); MA Abdelaziz, A Gado, U Hantour, AM Ibrahim, K Ibrahim (Al-Hussein Hospital, Egypt); M Abd-Elmawla, M Abdelkader, MS Aboul-Naga, N Adam, LAM Ahmed, M Alkelani, M Allam, MH Alnaby, A Assal, M Ebidy, MM Ebidy, NH El Gendy, RA El-Din, KH Elbisomy, AH Elgendy, AA Elrazek, A Elsawy, AA Elsharkawy, M Fahim, MF Hamed, AB Hassanein, A Ismail, M Ismail, M Karkeet, M Mabrouk, E Magdy, MI Mahmoud, R Mamdouh, ME Moghazy, M Mohamed, B Mowafy, M Nazir, HAG Shakshouk, M Shalaby, M Sleem, D Zahran (Alexandria Main University Hospital, Egypt); S Abdelhady, MR Aboelsoud, I Adel, H Ahmed, N Anwar, O Arafa, YH Asar, SA Awad, N Elsabbagh, FA Elsherif, M Gadelkarim, S Gamal, O Ghoneim, E Hany, O Hesham, K Hilal, A Hossameldin, M Ibrahim, EM Morshedy, ME Omar, AHEF Rida, R Saad, M Salama, M Salem, N Soliman (Alexandria Medical Research Institute, Egypt); A Aamer, AM Abdelraouf, M Abdelshakour, MG Azizeldine, KA Bassit, A Dahy, A Hasan, A Hashim, A Ibrahim, B Mahmoud, MA Mahmoud, B Mohamed, M Qenawy, AM Rashed, MM Saad, FA Sabour, F Sayed, M Sayed, AW Shamsedine, M Shawqi (Assiut University Hospital, Egypt); A Attia, KS El-Dien, A Shwky (Bab El-Shareia University Hospital, Egypt); SM Abdel-Kader, M Abdelaty, H Abdulaziz, EM Abdulhakeem, N Abdullah, A Abouzaid, M Abubakr, S Alaael-Dein, E Ali, HAA Amin, IM El Sayed, SA El-Din, EA Eldeen, MAB Eldin, AAE Elhusseiny, NAR Elsayed, M Elshaar, D Gamil, E Hashad, AAF Ibraheem, MK Ismail, MH Madkor, H Magdy, SME Mahmoud, S Mansour, AR Mohamed, F Mohamed, MA Mohamed, MT Ramadan, A Reda, A Refaat, M Saami, OM Salah, MM Salem, MY Shawky, NA Soliman, F Sroor, M Talaat, A Tarek, M Zakaria, MR Loaloa (Benha Faculty Of Medicine, Egypt); S Ahmed, A Ali, M Badawy, N El-Sagheer, A Essam, D Gamal, S Magdy, A Salah, M Salah (Beni Suef University Hospital, Egypt); A Abdelaal, A Aglan, S Ali, A Ata, AKZ Darwish, M El Halawany, E El-Gizawy, A Elazab, S Elhadry, E Elhalawany, S Elmihy, M Essam, A Farag, H Hajeh, O Moussa, M Nashat, M Nasr, A Rezq, AE Sallam, M Samy, M Samy, A Sheta, S Soliman, S Tariq, A Zohair (El-Menshawy General Hospital, Egypt); A Abdel-Aty, R Abdelhamed, O Abdelkader, K Ashour, E El-Taher, A Elhadad, SAM Farouk, S Ghanem, A Hassaan, EM Ibrahim, SM Matter, A Mohamed, I Rakha, Y Soliman, D Tarek (Faculty Of Medicine Seuz Canal University, Egypt); AR Abdelazeam, A Adelshone, AB Adnan, D Al-Marakby, CDM Ali, M Amreia, AY Ata, S Bahar, ERM Basir, A Elhendawy, MB Hasnan, MJB Ismail, SNA Kamarulzamil, A Latif, MAA Lokman, AHHA Majid, M Salma, S Shaharuddin, A Zulkifli (Faculty Of Medicine, Tanta University, Egypt); K Abdelbadeai, A Abdelfatah, MA Abdullah, H Ahmed, Y Allam, S Arafa, NM Badwi, AA El Dahab, A El-Sehily, N Elfouly, Y Elfouly, G Elhoseny, EA Elkhalek, E Ezzat, T Ezzat, AM Fathy, A Fergany, A Hassaan, ATA Hassan, OMM Hassan, A Ibrahim, A Ibrahim, EA Kasem, M Kelany, M Magdy, A Mohamed, AR Mohammed, MM Mohammed, S Mohammed, A Reda, AG Saad, HA Saad, AS Sleem, Y Zakaria (Faculty Of Medicine, Zagazig University, Egypt); G Abdelazim, I Abdelmotaleb, AK Abdrabou, M Aboelella, O Aboelmagd, BE Adel, A Ahmed, S Ahmed, A Al Meligy, K Alhady, MYM Aly, HM Bakry, M Bassem, AH Bekhet, NM Bekhet, K Dabbour, K Dawood, A El Kashash, NKA El-Latif, NM Elhadary, MS Elhelbawy, SS Elkholy, A Elnagar, MA Elnajjar, AA Elsameea, S Elsherbiney, N Elzahed, H Emadeldin, AA Essam, S Gaafar, A Gad, MO Gad, A Geuoshy, M Hafez, S Hafez, W Hamsho, D Hasan, I Hassan, R Husseiny, SA Ismail, AM Kandil, A Magdy, ME Maher, H Mahmoud, S Mahmoud, N Maraie, O Mattar, N Mesbah, SR Mohamed, H Saad, A Sabe, AK Sabe, M Saeed, AA Saleh, N Semeda, A Shahine, A Soliman, BA Tawfik, N Wael, E Zakaria (Kasr Al-Ainy Faculty Of Medicine, Cairo University, Egypt); E Abdallah, N Abdel-Hameed, A Denewar, R Elashry, H Elfeki, E Emara, S Emile, A Ghanem, M Mostafa, MFW Omar, E Rashad, A Sakr, A Sanad, G Tawfik, W Thabet, M Youssef, A Zaki (Mansoura University Hospital, Egypt); E Abdelmageed, DM Abdelrouf, EA Al Raouf, ES Elbanby, A Elfarargy, M Elgheriany, S Elhamouly, M Elmasry, E Elwy, A Esam, MM Farahat, E Gamal, H Gamal, A Hammad, EM Hegazy, E Ibrahim, H Kandil, T Khafagy, S Khallaf, EY Mansor, M Moaty, AM Mohamed, AE Mohammed, A Moustafa, GS Nagy, A Saidbadr (Menofiya University Hospital, Egypt); MM Eid, M Eldafrawy, AZ Eldeeb (October 6 University Hospital, Egypt); AAR Al Rafati, MFM Badr, A Bakr, A El-Sawy, R Elsemelawy, M Mostafa (Smouha University Hospital, Egypt); SM Al Attar, MA Badenjki, A Soliman (The Memorial Soaad Kafafi University Hospital, Egypt); A Reinsoo, S Saar, P Talving (The North Estonia Medical Centre, Estonia); A Fitsum, N Seyoum, T Worku (Addis Ababa University, College Of Health Sciences, School Of Medicine, Ethiopia); A Leppäniemi, V Sallinen, M Tolonen (Helsinki University Hospital, Finland); X Delforge, E Haraux (CHU Amiens Picardie, France); A Mariani, G Podevin, F Schmitt (CHU Angers, France); A Haffreingue, J Marret, J Rod (CHU Caen, France); N Bustangi, M Lopez, A Scalabre (CHU De Saint Etienne, France); J Bréaud, P Gastaldi, J Lecompte (CHU Lenval Nice, France); Q Ballouhey, L Fourcade, C Grosos (CHU Limoges, France); T Cecilia, F Helene, C Jean-Francois (CHU Martinique, France); MG Grella (CHU Poitiers, France); AP Arnaud, E Courboin, J Hascoet, B Maillot (CHU Rennes, France); M Renaux-Petel (CHU Rouen, France); O Abbo, AA Kaci, T Prudhomme (CHU Toulouse, France); B Dousset, S Gaujoux, R Schiavone (Cochin - APHP, France); S Dardenne, E Robert (GHICL, France); A Broch, E Hervieux, C Muller (Hopital Necker Enfants Malades, APHP, France); E Anis, R Claire, C Taieb (Hopital Robert Ballanger, Paris, France); S Irtan, B Parmentier, M Peycelon (Trousseau Hospital, APHP, France); E Akatibo, M Ekow, M Yakubu (Baptist Medical Center, Nalerigu, Ghana); FE Gyamfi (Berekum Holy Family Hospital, Ghana); ET Atkins, CL Coompson (Brongho-Ahafo Regional Hospital, Sunyani, Ghana); M Amoako-Boateng, M Dayie, S Debrah, R Hagan (Cape Coast Teaching Hosital, Ghana); E Ackom, E Akoto, E Mensah (Dormaa Presbyterian Hospital, Ghana); P Kwakyeafriyie (Essumejaman Sda Hospital, Dominase, Ghana); K Asare-Bediako, HEK Kordorwu, E Tackie (Keta Hospital District Hospital, Keta, Ghana); N Adu-Aryee, J Amoako, W Appeadu-Mensah, A Bediako-Bowan, W Bonney, J Clegg-Lampety, J Dakubo, F Dedey, S Essoun, V Etwire, H Glover-Addy, M Ohene-Yeboah, S Osei-Nketiah (Korle Bu Teaching Hospital, Ghana); K Agbedinu, M Amoah, C Dally, A Gyedu, A Yifieyeh (Kwame Nkrumah University of Science and Technology/Komfo Anokye Teaching Hospital, Ghana); PT Amoako (Samapa Government Hospital, Ghana); E Dagoe (St. Mary's Hospital, Ghana); F Owusu (St. Particks Hospital, Ghana); F Abantanga, EK Appiah, H Asumah, D Bandoh, ATT Kojo, M Kyereh, S Tabiri, P Wondoh (Tamale Teaching Hospital, Ghana); K Aaniana, E Acquah, A Avoka, K Kusi, K Maison, R Opoku-Agyeman (Techiman Holy Family Hospital, Ghana); V Dassah, A Davor (Upper East Regional Hospital, Ghana); S Abdul-Latif, GN Barnabas (Upper West Regional Hospital, Ghana); G Gkiokas, A Papailia, T Theodosopoulos (2nd Dept Of Surgery, Aretaieion Hospital, National & Kapodistrian University Of Athens School Of Medicine, Greece); O Ioannidis, D Kyziridis, S Parpoudi (4th Surgical Department, Aristotle University Of Thessaloniki, General Hospital, Papanikolaou, Greece); A Bamicha, D Lytras, K Psarianos (Achillopoyleio General Hospital Of Volos, Greece); G Gemenetzis, S Parasyris (Attikon University Hospital, Greece); K Farmakis, T Feidantsis, M Mitroudi, C Panteli (G. Gennimatas General Hospital, Greece); I Patoulias, D Sfougaris, I Valioulis (G. Gennimatas Hospital, Greece); G Karabelias, G Kyrou, I Papaskarlatos (General And Oncological Hospital Of Kifissia-Athens, Greece); S Germanos, K Konstantina, N Zampitis (General Hospital Of Larissa, Greece); A Stefanopoulos (General Hospital Of Nafplio, Department Of Surgery, Greece); C Agalianos, C Barkolias, C Ferousis, N Ivros, V Kalles, I Kyriazanos, A Tselos, G Tzikos, E Voulgaris (Naval And Veterans Hospital, Greece); D Balalis, D Korkolis, DK Manatakis (Saint Savvas Cancer Hospital, Greece); C Anthoulakis, M Margaritis, N Nikoloudis (Serres General Hospital, Greece); M Aguilera-Arevalo, O Coyoy-Gaitan, J Rosales (Hospital General San Juan De Dios, Guatemala); DM Cohen, A Matheu, GS Rosenberg (Hospital Herrera Llerandi, Guatemala); DH Cruz, CP Galvez, STT Rodriguez (Hospital San Vicente, Guatemala); E Barrios, R Soley, L Tale (Juan Josè Arevalo Bermejo, Guatemala); A Charles, M Paul (Hopital Universitaire De Mirebalais, Haiti); TK Chan, YHE Cheung, W Dao, CYJ Fok, SH Kwok, AC Lai, JCY Lam, WH Lam, TSB Lee, KW Leung, KHG Li, TWC Mak, YK Ng, HY Wong, MHA Yeung (Prince Of Wales Hospital, Hong Kong SAR, China); CC Foo, Q Liu, J Yang (University Of Hong Kong, Hong Kong SAR, China); S Kumar (Excelcare Hospital, India); P Alexander, N Aruldas (Lady Willingdon Hospital, India); W Dar, KC Janardha, U Muddebihal (Manipal Hospital, India); A Bhatnagar, B Kumar, V Upadhyaya (Sanjay Gandhi Post Graduate Institute Of Medical Sciences, India); FJ Adella, F Iskandar, AS Rulie, J Setiawan (Atma Jaya Hospital, Indonesia); CV Evajelista, H Natalie, A Suyadi (Dr. Oen Surakarta Hospital, Indonesia); N Adhitama, FFA Andika, HM Arsyad, R Gunawan, A Hasanah, H Karismaningtyas, LPS Mata, ADF Mukin, HD Nurqistan, NA Purwaningsih, DF Rahmah, TA Widiastini (Rsd Dr Soebandi, Indonesia); R Amandito, M Billy, A Clarissa, PA Gultom, A Haloho, WS Jeo, N Johanna, F Lee, N Sutandi (Rsupn Cipto Mangunkusumo, Indonesia); M Alherz, KC Conlon, RMNR Dorani, M Glynn, W Goh, HA Shiwani, L Sproule (Tallaght Hospital, Trinity College Dublin, Ireland); M Bala, A Kedar (Hadassah Hebrew University Medical Center, Israel); A Armellini, D Chiesa, G Pata (A.O. Spedali Civili Di Brescia, Italy); L Ansaloni, F Coccolini, GE Nita, E Vicario (AO Papa Giovanni XXIII, Italy); G Confalonieri, G Pesenti (Azienda Ospedaliera Alessandro Manzoni, Italy); B Brunoni, A Rinaldi, MN Ringressi (Azienda Ospedaliera Universitaria Careggi, Italy); L Bortolasi, T Campagnaro, S Conci, A Gulielmi, C Iacono, G Lazzari, S Manfreda, U Tedeschi, P Violi (Azienda Ospedaliera Universitaria Integrata di Verona, Italy); E Ciccioli, E Goldin, E Vendramin (Azienda Ospedaliera di Padova, Italy); F Aquilino, N Chetta, A Picciariello (Azienda Ospedaliero Universitaria Consorziale Policlinico Di Bari, Italy); D Andreotti, L Gavagna, S Occhionorelli, S Targa, G Vasquez (Azienda Ospedaliero-Universitaria Di Ferrara, Italy); SMM Basso, A Bigaran, A Favero (Azienda Per L'assistenza Sanitaria N. 5 Friuli Occidentale, Italy); M Migliore, S Mochet, M Perino, F Riente, P Salusso, D Sasia (Azienda Sanitaria Ospedaliera San Luigi Gonzaga, Italy); G Clerico, G Gallo, M Trompetto (Department of Colorectal Surgery, S. Rita Clinic, Vercelli, Italy); M Papandrea, R Sacco, G Sammarco (Department of Medical and Surgical Sciences, Policlinico Universitario Mater Domini Campus Salvatore Venuta, Catanzaro, Italy); L Bucci, MC Giglio, G Luglio, G Pagano, R Peltrini, V Sollazzo (Federico II University Of Naples, Italy); M Foco, FR Giardino, D Gui, G Perrotta, M Ripa (Fondazione Policlinico Universitario Agostino Gemelli, Italy); S Pasquali, A Simioni, D De Boni (IOV - Istituto Oncologico Veneto, Italy); L Bonavina, V Lazzari, Y Macchitella (IRCCS Policlinico, University of Milano, San Donato, Italy); M Abdelkhalek, A Belli, S De Franciscis (Istituto Nazionale Tumori Fondazione, Pascale-I.R.C.C.S., Italy); A Birindelli, G Tugnoli, S Di Saverio (Maggiore Hospital, Italy); P Mingrone, MA Paludi, D Pata (Nicola Giannettasio Hospital, Rossano, Italy); S Basilicò, C Corbellini, D Merlini (Ospedale Di Rho - ASST Rhodense, Italy); A Bondurri, N Leone, A Maffioli (Ospedale Sacco, Italy); P Aonzo, G Curletti, R Galleano (Ospedale Santa Corona, Pietra Ligure, Italy); AL Brocca, G Cocorullo, N Falco, T Fontana, L Licari, M Mangiapane, G Salamone, V Silvestri, R Tutino, P De Marco (Policlinico Paolo Giaccone di Palermo, Italy); C Arcudi, M Shalaby, P Sileri (Policlinico Tor Vergata Hospital, Rome, Italy); D Angelieri, A Antoniozzi, CD Basso, M Catani, D Coletta, M Coletti, N Depalma, F Falaschi, I Iannone, M Malavenda, A Natili, C Reali, S Ribaldi, D Rossi (Policlinico Umberto I Emergency Surgery Department, Italy); S Berti, S Boni, E Francone (S. Andrea Hospital, Poll-Asl 5, La Spezia, Italy); A Benevento, L Giavarini, F Pata (Sant'Antonio Abate Hospital, Gallarate, Italy); G Balducci, AL Conte, L Lorenzon (Sant'Andrea Hospital, Sapienza University of Rome, Italy); F Bianco, F Steccanella, L Turati (Treviglio Hospital, Italy); G Pellino, F Selvaggi, L Selvaggi, N Di Martino (Universitá della Campania “Luigi Vanvitelli”, Naples, Italy); A Ababneh, L Abusalem, E Al-Dakka, K Aljboor, A Alnusairat, I Bsisu, O Halhouli, A Qaissieh (Jordan University Hospital, Jordan); H Mohammed, T Yusufali (Kenyatta National Hospital, Kenya); J Lando, W Ndegwa, R Parker (Tenwek Hospital, Kenya); D Dragatas, P Višinskas, R Žilinskienė (Hospital Of Jonava, Lithuania); A Dulskas, J Kuliavas, NE Samalavicius (Klaipeda University Hospital, National Cancer Institute, Lithuania); J Gribauskaite, M Jokubauskas, D Venskutonis (Lithuanian University Of Health Sciences, Lithuania); S Bradulskis, E Dainius, Z Dambrauskas, A Gulbinas, T Jankus, K Jasaitis, S Kasputyte, M Kiudelis, D Mikuckyte, M Montrimaite, V Nevieraite, A Parseliunas, S Petrikenas, R Riauka, E Slapelyte, A Subocius, L Venclauskas, J Zilinskas (Lithuanian University of Health Sciences, Lithuania); N Kaselis, G Žiubrytė (Republican Hospital Of Klaipeda, Lithuania); M Pažuskis, Z Urniežius, M Vilčinskas (Republican Hospital of Kaunas, Lithuania); A Burmistrovas, Z Tverskis (Taurage Hospital, Lithuania); R Mazelyte, A Vaicius, A Zadoroznas (Viesoji Istaiga Rokiskio Rajono Ligonine, Lithuania); T Abaliksta, VJ Banaitis, D Danys, M Drungilas, V Gaižauskas, E Grisin, V Jotautas, A Ladukas, K Lagunavicius, E Laugzemys, V Lipnickas, D Majauskyté, P Mazrimas, S Mikalauskas, T Poškus, R Rackauskas, G Simutis, EZ Sruogiene, L Uščinas (Vilnius University Hospital, Lithuania); FCFP Rahantasoa, F Rasoaherinomenjanahary, LH Samison, TEC Tolotra (Joseph Ravoahangy Andrianavalona Hospital, Madagascar); C Kwatiwani, N Msiska, V Msosa, C Mukuzunga (Kamuzu Central Hospital, Malawi); SMD Asilah, FY Chai, K Gunaseelan, WN'WM Nasir, KZ Syibrah, P Yoganathan (Hospital Keningau, Malaysia); PY Koh, EX Lee, SY Lim, JE Saw, SY Teo, LJ Yeang (Hospital Pulau Pinang / Penang Medical College, Malaysia); YY Gan, JRS Ting (Hospital Sibu, Malaysia); AEZ Cheah, CYN Chow, Y Der, PAL Har, KL Koay, TNT Mat, SSY Sii, YK Tan, CY Wong (Hospital Sultanah Aminah, Malaysia); YJ Cheong, C Gan, HE Heng, SN Kong, YT Mok, YT Neo, K Palayan, YW Tan, MD Tata (Hospital Tuanku Ja'afar, Malaysia); PX Chin, NZ Riswan, A Salleh (Kajang Hospital, Malaysia); NAN Abdullah, SAWEW Ali, KJ Chung, DL Jethwani, R Julaihi, SW Mathew, MK Nirumal, RGE Tze, MT Yahaya (Sarawak General Hospital, Malaysia); F Henry, X Low, YY Tew (Selayang Hospital, Malaysia); DNA Aziz, NR Kosai, R Rajan, MM Taher (Universiti Kebangsaan Malaysia Medical Centre UKMMC, Malaysia); NA Aziz, C Chai, H Chong, S Kumar, K Poh, AC Roslani (University Malaya Medical Centre, Malaysia); I Bertuello, K Bonavia, E Borg, SD Brincat, GM Camilleri, K Carabott, K Cassar, J Dalli, T Dimech, M Falzon, A Farrugia, N Grech, T Grima, VTH Le, D Magri, C Mizzi, S Mizzi, A Navarro, K Sammut, R Scicluna, N Shaikh, T Tembo, S Zammit, C Zarb (Mater Dei Hospital, Malta); S Corro-Diaz, M Manriquez-Reyes, A Ramos-De la Medina (Hospital Español de Veracruz, Mexico); M Abbouch, A Abdelhamid, H Bachri, A Belkouchi, S Benammi, RM Bennai, C Benyaiche, K Boukhal, A Hrora, MS Jabal (IBN Sina Hospital, Morocco); L Duinhouwer, M Vermaas (Ijsselland Hospital, Netherlands); MS Merlo, J Pastora, G Wood (Hospital Escuela Oscar Danilo Rosales Arguello, Nicaragua); A Adamu, H Aliyu, M Aliyu, S Aliyu, S Baba, M Daniyan, O Ogunsua, T Sholadoye, Y Ukwenya (Ahmadu Bello University Teaching Hospital Zaria, Nigeria); L Anyanwu, A Mohammad, A Sheshe (Aminu Kano Teaching Hospital, Nigeria); O Adebola, A Adesina, O Faturoti, O Odutola, C Onuoha, O Taiwo (Babcock University Teaching Hospital, Nigeria); J Ajah, S Kache, J Makama (Barau Dikko Teaching Hospital, Kaduna State University, Kaduna, Nigeria); O Abiola, A Adeyeye, A Ajiboye, I Amole, A Olaolorun (Bowen University Teaching Hospital, Nigeria); A Adebanjo, A Adeniyi, O Adesanya (Federal Medical Centre, Abeokuta,, Nigeria); O Ajai, F Balogun, I Njokanma, M Oludara, R Osuoji, O Williams (Lagos State University Teaching Hospital, Nigeria); A Ademuyiwa, B Adenekan, F Alakaloko, C Bode, O Elebute, G Ihediwa, A Lawal, V Nwinee, TO Olajide, M Olugbemi, O Oshati, A Osinowo (Lagos University Teaching Hospital, Nigeria); A Abdurrazzaaq, A Ajao, O Ayandipo, T Lawal (University College Hospital, Ibadan, Nigeria); P Mshelbwala, B Odeyemi, S Olori, G Samson, SA Samuel, OK Timothy (University Of Abuja Teaching Hospital,, Nigeria); J Adeniran, A Adeyeye, M Alada, O Habeeb, A Nasir, A Popoola (University Of Ilorin Teaching Hospital,Ilorin, Nigeria); B Bello, H Mendel, U Muktar (Usmanu Danfodiyo University Teaching Hospital, Nigeria); KM Augestad, GS Banipal, TT Moe, M Monteleone, JK Schultz (Akershus University Hospital, Norway); T Gaarder, PW Monrad-Hansen, PA Næss (Oslo University Hospital, Norway); R Herikstad, A Kanani, JW Larsen, K Styles, JA Søreide, K Søreide, T Veen (Stavanger University Hospital, Norway); S Holte, G Lauzikas, J Wiborg (Sykehuset Telemark HF, Norway); EK Aahlin, M Gran, E Jensen (University Hospital Of Northern Norway, Norway); J Abbasy, AR Alvi, T Gala, N Shahzad (Aga Khan University, Pakistan); N Nadeem, M Saqlain (Allied Hospital, Faisalabad, Pakistan); A Ahmed, KF Bhopal, MT Butt, Z Iftikhar, AK Niazi, SAU Razi (Bahawal Victoria Hospital, Pakistan); M Javaid, MA Khan, M Waqar (CMH Lahore Medical And Dental College, Pakistan); M Adil, F Baluch, A Bani-Sadar, AU Qureshi, A Raza, A Raza, I Raza (King Edward Medical University, Mayo Hospital, Lahore, Pakistan); M Amjad, MM Arshad (Nishtar Medical College And Hospital, Pakistan); S Abushamleh, T Al-taher, A Hamarshi, A Hamdan, S Hanoun, D Jaradat, A Musleh, AA Qumbos, R Saadeh, A Salman, AA Taher (Al Makassed Islamic Charitable Society Hospital, Jerusalem, Palestine); H Al-farram, S Al-saqqa, I Awad, A Bowabsak, A El Jamassi, A Firwana, M Hamdan, D Hasanain, M Salah (Al-Shifa Hospital, Palestine); M Altarayra, M Ghannam, A Herebat, I Qawasmi, K Qurie, A Shaheen (Alia Governmental Hospital, Palestine); I Adawi, M Adawi, A Elmashala (Beit Jala Governmental Hospital, Palestine); FE Al Barrawi, A Ashour (Bit Hanoun Hospital, Palestine); A Ghaben, A Ashour (Indonesian Hospital, Gaza, Palestine); Y Abuowda, S Afana, A Al-Buhaisi, E Alaloul, S Alyacoubi, H Baraka, M Elshami, S Jaber, J Meqbil (Islamic University of Gaza Medical School, European Gaza Hospital & Shifa Hospital, Palestine); R Khreishi, R Khreishi (Martyr Thabet Govermental hospital, Tulkarem, Palestine); E Abuqwaider, T Idress (Mizan hospital, Palestine); M Al-faqawi, A Al-khatib, M Fares (Nasser Hospital, Palestine); A Abdelhaq, M Abu-toyour, F Asi, A Atiyeh, M Dabboor, M Mustafa, A Shalabi, A Shamasneh, R Zaa'treh (Palestine Medical Complex, Palestine); JT Cardozo, RAM Cardozo, HAS Lohse, LIP Lopez, MO Roche, GRP Servin, GMM Vega (Hospital de Clínicas, II Cátedra de Clínica Quirúrgica, Universidad Nacional de Asunción, Paraguay); J Salcedo, R Velasquez (Clínica De Especialidades Médicas, Peru); AMS Barrantes, JAC Bravo, CG Dueñas, KT Espinoza, C Fernández, L Fuentes-Rivera, B Málaga, D Romani, S Shu, J Ye (Hospital Cayetano Heredia, Peru); LAM Barrientos, ESF Farfan, JLH Hamaguchi, JJH Matta (Hospital De Policia, Peru); A Robledo-Rabanal, LAZ Solis, AJR Velásquez (Hospital III José Cayetano Heredia, Peru); YEA Bermúdez, AC Calua, J Carpio, N Carrasco, F Espinoza, HS Miyasato, PAT Orbegozo, N Ortiz, WR Panez, C Razuri, X Rodriguez, ADP Rojas, CS Samaniego, D Sanchez, F Saravia, SG Torres, M Valcarcel-Saldaña (Hospital Nacional Arzopispo Loayza, Peru); AL Contreras-Vergara, AGV Mejia, MSG Montejo (Hospital Nacional Guillermo Almenara Irigoyen, Peru); KT Espinoza (Hospital Nacional Maria Auxiliadora, Peru); R Mas, ADP Paucar, MDCE Salas, GCM Sila, WA Ticona, M Vargas (Hospital Regional De Ayacucho, Peru); CL Almanon, MC Lapitan, MD Parreno-Sacdalan (Department Of Surgery, Philippine General Hospital, University Of The Philippines Manila, Philippines); MJB Maño, JJV Mora, MAP Redota, MF Roxas (The Medical City, Philippines); A Lasek, P Major, D Radkowiak, M Rubinkiewicz (2nd Department Of Surgery, Jagiellonian University Medical College, Poland); M Janik, R Roszkowski, M Walędziak (Department Of General, Oncological, Metabolic And Thoracic Surgery, Military Institute Of Medicine, Warsaw, Poland); J Costa-Maia, C Fernandes, R Melo (Centro Hospitalar De São João, Portugal); M Beuran, MR Bratu, C Ciubotaru, B Diaconescu, I Negoi, M Vartic (Emergency Hospital of Bucharest, Romania); A Kourdouli, M Popa (Spital Judetean De Urgenta Din Craiova, Romania); AS Mironescu, L Muntean, LC Vida (Spitalul Clinic De Copii Brasov, Romania); H Mircea (Spitalul Clinic Judetean Brasov, Romania); D Duhoranenayo (Kibungo Hospital, Rwanda); JCA Ingabire, AZ Mutabazi, N Uzabumwana (University Teaching Hospital Of Kigali, Rwanda); E Jovine, G Landolfo, N Zanini (San Marino State Hospital, San Marino); MSA Alghamdi, M Aljiffry, A Alkaaki, A Altaf, F Idris, A Khoja, A Maghrabi, A Nawawi, S Turkustani (Department of Surgery, Faculty of Medicine, King Abdulaziz University Hospital, Jeddah) Eyad Khalifah, Ahmad Gudal, Adel Albiety, Sarah Sahel, Reham Alshareef, Mohammed Najjar, Saudi Arabia); F Al Bastawis, N Al Subaie, B Alhassan, A Altamimi, A Altamimi, R Alyahya, M Hassanain, R Khan, T Nouh (King Khaled University Hospital, Saudi Arabia); L Jeremic, M Nestorovic, M Radojkovic (Clinic For General Surgery, Clinical Center Nis, Serbia); XW Chan, CS Chong, LWL Joel, S Koh, JH Law, KY Lee, KC Lee, FQH Leong, B Lieske, JK Tan, KSK Tan, RCK Tan (National University Hospital, Singapore); N Maistry (Charlotte Maxeke Johannesburg Academic Hospital, South Africa); V Jennings, A Leusink, R Moore (Chris Hani Baragwanath Academic Hospital, South Africa); ME Mabitsela, SR Ndlovu (Dr George Mukhari Academic Hospital, South Africa); V Kong (Edendale Hospital, South Africa); J Joosten, J Pape, L Roodt, A Sander, S Sobnach, R Spence (Groote Schuur, South Africa); S Rayne, S Van Straten (Helen Joseph Hospital, University Of Witwatersrand, South Africa); F Anderson, T Madiba, Y Moodley (Inkosi Albert Luthuli Central Hospital, South Africa); K Kinandu, P Ndwambi, M Tun (Leratong Hospital, South Africa); F Du Plooy (Mediclinic Potchefstroom, South Africa); M Badicel, R Jaich (Milpark Hospital, South Africa); G Chilton, L Hartford, P Karjiker (Mitchell's Plain District Hospital, South Africa); H Bougard, K Chu, A Dell, J Gouws, N Kariem, F Noor (New Somerset Hospital, South Africa); K Kabongo, A Khamajeet, SK Tshisola (Stanger Hospital, South Africa); S Burger, Q Ellison, DC Grobler, LB Khulu, F Du Toit (Tembisa Tertiary Provincial Hospital, South Africa); B Dedekind, MI Hampton, P Nashidengo, K Pluke (Victoria Hospital Wynberg, South Africa); CG Bernardo, E Contreras, A Dorismé, LS García, J Pagnozzi, J Rodicio, S Sanz, J Stuva, A Suarez, TD Vico, AM De León (Central University Hospital Of Asturias, Spain); L Garcia-Florez, JL Otero-Díez, VR Pérez, NA Suárez (Hospital Universitario San Agustín, Spain); D Ambrona-Zafra, A Craus-Miguel, P Diaz-Jover, L Fernandez-Vega, JM Garcia-Perez, P Jimenez-Morillas, A Mazzella, C Pineño-Flores, N Pujol-Cano, JJ Segura-Sampedro, F Sena-Ruiz, C Soldevila-Verdeguer (Hospital Universitario Son Espases, Spain); VJ Carneros, MV Collado, JM García, SC Moreno, JG Septiem (Hospital Universitario de Getafe, Spain); V Andriola, R Blanco-Colino, E Espin-Basany (Hospital Valle De Hebron, Spain); E Esteban, E Ferrero, M Gonzalez, I Ortega, A Picardo (Infanta Sofía University Hospital, Spain); J Ruiz-Tovar (University Hospital Rey Juan Carlos, Spain); AB Jayathilake, SPB Thalgaspitiya, LS Wijayarathna, PMS Wimalge (University Surgical Unit, Teaching Hospital Anuradhapura, Sri Lanka); A Ndajiwo, O Okenabirhie, HA Sanni (Joseph N France Hospital, St. Kitts And Nevis); M Abdulaziz, A Adam, A Homeida, A Mussad, OA Omer, A Younis (University of Gezira, Sudan); M Hjertberg (Department Of Surgery And Department Of Clinical And Experimental Medicine, Linköping University, Norrköping, Sweden); A Thorell, F Wogensen (Ersta Hospital, Sweden); H Thorarinsdottir (Helsingborgs Lasarett, Sweden); P Elbe, L Forlin, W Rutkowski, D Saraste (Karolinska Universitetssjukhuset, Solna, Sweden); M Breistrand, A Sokratous (Mora Hospital, Sweden); S Ahlqvist, S Ahlqvist, I Björklund, Y Cengiz (Sundsvall Hospital, Sweden); K Niska, M Sund (Umea University Hospital, Sweden); A Chabok, M Nikberg, J Sigurdadottir (Västmanlands Hospital Västerås, Sweden); R Schmid, G Werder (Buergerspital Solothurn, Switzerland); R Bluelle, D Frey, D Oswald, A Palma, G Peros, K Reinisch, G Zuk (Gzo Spital Wetzikon, Switzerland); A Gübeli, J Müller, LW Widmer (Hospital Davos, Switzerland); A Gerosa, S Mahanty, A Nocito (Kantonsspital Baden, Switzerland); DA Raptis, M Zuber, L Zumbühl (Kantonsspital Olten, Switzerland); C Adıyaman, S Bayram, TB Cengiz, M Cevik, V Işler, BB Kobal, D Mutlu, V Ozben, BB Ozmen, AM Pektaş, I Sapci, I Tansoker, ÖF Toto, S Yolcu, HC Çakaloğlu (Acibadem University School of Medicine, Atakent Hospital, Turkey); Y Altinel, OB Gulcicek, T Vartanoglu (Bagcilar Research And Training Hospital, Turkey); H Alis, I Halicioglu, NA Sahbaz (Bakirkoy Dr. Sadi Konuk Training And Research Hospital, Turkey); E Arslan, BE Baki, S Bodur, S Celik, A Guner, E Gül, B Murutoglu, A Semiz, K Tomas, R Yildirim (Karadeniz Technical University Faculty Of Medicine, Turkey); MC Aydin, SR Karahan, E Kose (Okmeydanı Training And Research Hospital, Turkey); K Karabulut, V Mutlu, BB Ozkan (Ondokuz Mayis University Medical Faculty, Turkey); KY Chen, R Heard, S Nanthakumaran (Aberdeen Royal Infirmary, United Kingdom); R Breslin, R Srinivasan (Blackpool Victoria Hospital, United Kingdom); A Boggon, K Connor, A Haslegrave, K Laurie, T Mann (Borders General Hospital, United Kingdom); E Dashnyam, E Kalakouti, A Mehdi, N Post, F Stourton, O Warren, R White (Chelsea And Westminster Hospital, United Kingdom); A Paramasivan (Darlington Memorial Hospital, United Kingdom); N Blencowe, K Bowling, D Bunting, P Ireland (Gloucestershire Royal Hospital, United Kingdom); E Reunis, WC Soon, R Tyler (Good Hope Hospital, United Kingdom); D Kufeji, C Skerritt, N Wright (Guy's And St. Thomas' Hospitals, United Kingdom); B Barmayehvar, U Datta, SK Kamarajah, S Karandikar (Heartlands Hospital, United Kingdom); L Dick, I Liew, NG Mairs, M Qureshi, A Rocke (Inverclyde Royal Hospital, United Kingdom); G Bond-Smith, N Farhangmehr, M Perenyei, T Pezas, T Urbonas (John Radcliffe Hospital, Oxford, United Kingdom); T Alhammali, AA Ibrahem, Y Salama (Kettering General Hospital NHS Trust, United Kingdom); MA Gani, G Gravante (Leicester Royal Infirmary, United Kingdom); MR Iqbal, A Jeffery, H Jeon, S Khosla, J Perera (Maidstone & Tunbridge Wells NHS Trust, United Kingdom); A Jeffery, J Perera (Maistone and Tunbridge Wells NHS Trust, United Kingdom); R Kabariti, S Oram (Nevill Hall Hospital, United Kingdom); S Chiu, F Cullen, T Kidd, C Owen, H Sarafilovic, M Wilson (Ninewells Hospital, United Kingdom); D Fouad, A Minocha (Norfolk And Norwich University Hospital, United Kingdom); S Kadiwar, J Luck, A Smedley (North Middlesex University Hospital, United Kingdom); C Currow, I Mykoniatis (Northampton General Hospital, United Kingdom); SI Tani (Nottingham University Hospital NHS Trust, United Kingdom); S Knight, D Nassif, A Sharma (Perth Royal Infirmary, United Kingdom); W Ali, T Dissanayake, A Ho, A Tennakoon (Pilgrim Hospital, United Lincolnshire Hospitals NHS Trust, United Kingdom); J Lim, JCK Ng (Queen Elizabeth University Hospital, Glasgow, United Kingdom); A Gupta, V Shatkar, F Wong (Queen's Hospital, BHR University Hospitals NHS Trust, United Kingdom); P Donnelly, E Monaghan, M Walker (Raigmore Hospital, United Kingdom); A Abbas, C Andress, C Bisset, YR Chin, E Evans, N Ishak, S Kamya, J Ploski (Royal Alexandra Hospital, Paisley, United Kingdom); J Blackwell, P Herrod, J Lund, R Wakefield (Royal Derby Hospital, United Kingdom); K Keogh, L Longstaff, N Smart (Royal Devon & Exeter Hospital, United Kingdom); YL Ang, J Camilleri-Brennan, MS D'Souza, DE Henshall, H Lim, K Mclean, S Mirza, ZH Ng, J Park, S Paterson-Brown, S Pronin, C Roy, L Tang, E Teasdale, EZ Ter, L Walls, S Yap (Royal Infirmary Of Edinburgh, United Kingdom); S Cole, N Shrimanker, M Stoddart, N Walker (Royal United Hospital Bath, United Kingdom); A Bandi, F Cohen, S Giuliani (St George's Hospital, United Kingdom); K Baillie, R Bamford, N Harvey, S Kershaw, L Nicholson, P Orton, M Palliyil, S Patel, S Shillito (Stockport NHS Foundation Trust, United Kingdom); T Abbott, O Akpenyi, H Caydiid, W English, E Hall, L Maciejec, S Mahdi, C Morgan, Z Rob, HD Torrance, D Townsend (The Royal London Hospital, United Kingdom); G Irwin, R Johnston (Ulster Hospital Dundonald, United Kingdom); D Chowdhury (University Hospital Ayr, United Kingdom); D Evans, P Patel (University Hospital, Wales, United Kingdom); R Davies, E Griffiths, A Mansuri, D Nepogodiev (University Hospitals Birmingham NHS Trust, United Kingdom); C Jones, SJ Lim, S O'Neill, C Tan (Victoria Hospital Kirkcaldy, United Kingdom); D Dhillon, GM Jama, K Patel (Walsall Manor Hospital, Walsall, United Kingdom); A Al-Bahrani, M Elshaer, K Hunter (Watford General Hospital, United Kingdom); S Dindyal, K Majid, S Rajmohan, C Smith (West Middlesex University Hospital, United Kingdom); L Chan, F Din, C Eng, A L'Heveder, S McGarvie, K McIntosh, EHG Park, R Ravishankar, AR Shahbaz, JD Yau (Western General Hospital, Edinburgh, United Kingdom); E Teasdale (Western Isles Hospital, United Kingdom); S Blacker, A Kaul, J Parakh (Whiston Hospital, United Kingdom); S Awadallah, S Farag, A Nessa (Worthing Hospital, Western Sussex Hospital NHS Foundation Trust, United Kingdom); M Beamon, C Caliman, T Duane (John Peter Smith Hospital, United States); A Choudhry, N Haddad, M Zielinski (Mayo Clinic, United States); K Gash, RP Kiran, A Murray (New York Presbyterian Hospital / Columbia University Medical Center, United States); R Narayanan, M Swaroop (Northwestern Memorial Hospital / Northwestern University, United States); R Deal, J Myers, E Schadde (Rush University Medical Centre, United States); M Hemmila, L Napolitano, K To (University Of Michigan Medical Center, United States); M Dasari, E Etchill, J Puyana (University Of Pittsburgh Medical Center - Presbyterian, United States); M Maimbo, A Makupe, J Musowoya (Ndola Central Hospital, Zambia); D Kumwenda, K Otten, M Prins, A Reece-Smith, N Van Der Naald, A Verbeek (St Francis Mission Hospital, Zambia)

**GlobalSurg-2 Local validators**: R Balmaceda (Hospital Lagomaggiore, Argentina); AAB Suarez (Simplemente Evita, Argentina); C Deane (Queen Elizabeth Hospital, Barbados); E Dijan (Zadar General Hospital, Croatia); M Elfiky (Kasr Al Ainy Faculty of Medicine, Cairo University, Egypt); L Koskenvuo (Helsinki University Hospital, Finland); P Buisson (CHU Amiens Picardie, France); N Henric (CHU Angers, France); J Rod (CHU Caen, France); B Limoges (CHU Limoges, France); O Rosello (CHU Nice, France); A Thollot (CHU Poitiers, France); O Azzis (CHU Rennes, France); J Leroux (CHU Rouen, France); S Etienne (CHU Saint Etienne, France); K Pinnagoda (CHU Toulouse, France); P Francois (GHICL, France); C Alexandre (Hopital Cochin, APHP, France); C Capito (Hopital Necker Enfants Malades, APHP, France); S Hmila (Hopital Robert Ballanger, Paris, France); H Kotobi (Trousseau Hospital, APHP, France); O Imoro (Baptist Medical Centre, Ghana); OE Abem (Komfo Anokye Teaching Hospital, Ghana); J Clegg-Lamptey (Korle Bu Teaching Hospital, Ghana); P Wondoh (Upper West Regional Hospital, Ghana); V Soulou (Anticancer Hospital Of Athens Agios Savvas, Greece); D Papageorgiou (Naval And Veterans Hospital Of Athens, Greece); L Peña (Hospital General San Juan De Dios, Guatemala); S Asturias (Hospital Herrera Llerandi Amedesgua, Guatemala); B Kumar (Sanjay Gandhi Post Graduate Institute Of Medical College Lucknow, India); DB O'Connor (Tallaght Hospital, Trinity College Dublin, Ireland); A Taddei (Azienda Ospedaliera Universitaria Careggi, Italy); A Ruzzenente (Azienda Ospedaliera Universitaria Integrata di Verona, Italy); M Notarnicola (Azienda Ospedaliero Universitaria Consorziale Policlinico Di Bari, Italy); G Pascale (Azienda Ospedaliero-Universitaria di Ferrara, Italy); P Ubiali (Azienda per L'Assistenza Sanitaria N. 5 ‘Friuli Occidentale’, Pordenone, Italy); E De Luca (Department of Medical and Surgical Sciences, Policlinico Universitario Mater Domini Campus Salvatore Venuta, Catanzaro, Italy); M Sacco (Federico II University of Naples, Italy); MM Pascale (Fondazione Policlinico Universitario ’Agostino Gemelli’, Italy); C Cona (IOV - Istituto Oncologico Veneto, Italy); G Rotunno (Nicola Giannettasio Hospital, Rossano, Italy); M Corbellino (Ospedale Luigi Sacco Milano, Italy); E Morandi (Ospedale di Rho – ASST Rhodense, Italy); V Guglielmo (Policlinico Umberto I, Emergency Surgery Department, Italy); E Muzio, NA (S. Andrea Hospital, Poll-Asl 5, La Spezia, Italy); P Mao (San Luigi Gonzaga Hospital, Orbassano, Italy); C Bottini (Sant'Antonio Abate Hospital, Gallarate, Italy); AR Luc (Santa Rita Clinic, Vercelli, Italy); T Bocchetti, NA (Sant'Andrea Hospital, Sapienza University of Rome, Italy); R Cautiero (Second University Of Naples, Italy); AA Russo (Treviglio Hospital, Italy); M Notarnicola (University Of Bari ‘Aldo Moro’, Italy); L Solaini (University Of Brescia, Spedali Civili Di Brescia, Italy); FM Ali (Jordan University Hospital, Jordan); J Kutkevicius (Department Of General Surgery, Lithuanian University Of Health Sciences, Lithuania); P Ignatavicius (Hospital Of Lithuanian University Of Health Sciences Kaunas Clinics, Lithuania); J Žilinskas (Klaipeda Republic, Lithuania); R Baltrunas (Rokiskis District Municipality Hospital, Lithuania); P Kondrotas (Taurage County Hospital, Lithuania); K Strupas (Vilnius University Hospital, Lithuania); JY Siaw (Hospital Sibu, Malaysia); CL Tan (Hospital Sultanah Aminah, Malaysia); SY Yam (Penang Medical College, Malaysia); L Wilson (Sarawak General Hospital, Malaysia); MRA Aziz (University Malaya Medical Centre, Malaysia); J Bondin (Mater Dei Hospital, Malta); CD Zorrilla (Hospital Espanol De Veracruz, Mexico); A Majbar (Centre Hospitalier Ibn Sina Rabat, Morocco); E Nwabuoku (Ahmadu Bello University Teaching Hospital, Nigeria); A Taiwo (Babcock University Teaching Hospital, Nigeria); D Sale (Barau Dikko Teaching Hospital, Nigeria); L Abdullahi (Kano Aminu, Nigeria); O Faboya (Lagos Lasuth, Nigeria); A Fatuga (Lagos Luth, Nigeria); O Osagie (University Of Abuja Teaching Hospital, Nigeria); M Bliksøen (Oslo University Hospital, Norway); ZA Khan (Bahawal Victoria Hospital, Bahawalpur, Pakistan); J Coronel (Hospital de Clínicas, II Cátedra de Clínica Quirúrgica, Universidad Nacional de Asunción, Paraguay); C Miranda (Hospital Nacional Cayetano Heredia, Peru); LM Helguero-Santin (Hospital Regional III Jose Cayetano Heredia – Piura, Peru); I Vasquez (Lima Almenara, Peru); A Mironescu (Spitalul Clinic De Copii Brasov, Romania); J Rickard (Centre Hospitalier Universitaire De Kigali, Rwanda); A Adedeji (Joseph N France Hospital, Saint Kitts and Nevis); S Alqahtani (King Fahad General Hospital, Saudi Arabia); MZ Koto (Dr George Mukhari Academic Hospital, South Africa); M Rath (Groote Schuur Hospital, South Africa); M Van Niekerk (New Somerset Hospital, South Africa); R Matos-Puig (Stanger Hospital, South Africa); L Israelsson (Sundsvall, Sweden); T Schuetz (Kantonsspital Olten, Switzerland); M Mericliler (Acibadem University School of Medicine, Atakent Hospital, Turkey); M Uluşahin (Karadeniz Technical University Farabi Hospital, Turkey); MA Yuksek (Ondokuz Mayis University, Turkey); MMH Farhan-Alanie (Inverclyde Royal Hospital, United Kingdom); N Redgrave (John Radcliffe Hospital, Oxford, United Kingdom); M Wilson (Ninewells Hospital And Medical School, United Kingdom); R Callan (North Middlesex University Hospital, United Kingdom); GL Yong (Perth Royal Infirmary, United Kingdom); K Lee (Queen Elizabeth Birmingham, United Kingdom); B Wolf (Raigmore Hospital Inverness, United Kingdom); CK Musyoka (Royal Alexandra Hospital, United Kingdom); M Cox (Royal Derby Hospital, United Kingdom); K Whitehurst (Royal Devon And Exeter, United Kingdom); C Fairfield (Royal Infirmary Of Edinburgh, United Kingdom); J Olivier (Royal United Hospital Bath, United Kingdom); C Chibuye (Ndola Central Hospital, Zambia)

## Supplementary Material

znae129_Supplementary_Data

## Data Availability

The data sets generated during and/or analysed during the present study are available from the corresponding author on reasonable request.
